# Constituents of *Chimaphila japonica* and Their Diuretic Activity

**DOI:** 10.3390/molecules29051092

**Published:** 2024-02-29

**Authors:** Yue Yu, Deri Hu, Jinze Liu, Chenghao Wu, Yuhong Sun, Mingyue Lang, Xuan Han, Dongzhou Kang, Jun Zhe Min, Hong Cui, Mingshan Zheng

**Affiliations:** 1 School of Pharmaceutical Sciences, Yanbian University, Yanji 133000, China; 16604588858@163.com (Y.Y.); 15149910329@163.com (D.H.); yxh209644689@163.com (J.L.); 2023010925@ybu.edu.cn (C.W.); sunyuhong6012@163.com (Y.S.); 15948504041@163.com (M.L.); kangdz@ybu.edu.cn (D.K.); junzhemin23@163.com (J.Z.M.); 2 School of Pharmaceutical Sciences, Jilin University, Changchun 130021, China; hanxuan199907@163.com; 3 Key Laboratory of Natural Medicines of the Changbai Mountain, Ministry of Education, Yanbian University, Yanji 133000, China; 4 Center of Medical Functional Experiment, Yanbian University College of Medicine, Yanji 133000, China

**Keywords:** *Chimaphila japonica*, phenols, cyclohexanol, flavonoids, diuretic activity, molecular docking

## Abstract

Three new phenols (**1**–**3**), one new cyclohexanol (**4**), two known phenols (**5**–**6**), and six known flavonoids (**7**–**12**) were isolated from the *n*-butanol of the 75% ethanol extract of all plants of *Chimaphila japonica* Miq. Among them, compound **5** was named and described in its entirety for the first time, and compounds **9** and **10** were reported in *C. japonica* for the first time. The structures of all compounds were confirmed using a comprehensive analysis of 1D and 2D NMR and HRESIMS data. Biological results show that compounds **4**, **7**, and **11** exhibited potent diuretic activity. The modes of interaction between the selected compounds and the target diuretic-related WNK1 kinase were investigated in a preliminary molecular docking study. These results provided insight into the chemodiversity and potential diuretic activities of metabolites in *C. japonica*.

## 1. Introduction

Diuretics play a crucial role in enhancing urine production and facilitating the excretion of water and electrolytes from the body, making them indispensable for the management of a wide spectrum of medical conditions, such as hypertension, congestive heart failure, kidney disorders, and certain edematous states [1,2]. Diuretics are classified into different types, such as loop, thiazide, potassium-sparing, and carbonic anhydrase inhibitors, based on their site of action in the kidneys [3]. Nevertheless, the utilization of these pharmacological agents is coupled with potential side effects or adverse consequences. Improper or excessive use of diuretics can lead to electrolyte and fluid loss, triggering compensatory mechanisms such as the renin–angiotensin system (RAS), which increases renal sodium retention throughout the nephron [4,5]. Therefore, there is an urgent need to develop alternative drugs that are more effective and have fewer side effects. With the growing understanding of the physiology of renal salt, water reabsorption, and their regulation, new possibilities have been spawned for diuretic development. With-No-Lysine kinase 1 (WNK1), a member of the serine/threonine kinase family, was first identified in 2000. It is characterized by the unusual location of lysine in kinase subdomain I, as opposed to subdomain II [6]. To date, WNK1 has been found to be involved in a wide range of physiological and pathological processes, particularly in the control of ion transport and electrolyte balance in the kidney [7,8]. The diuretic impact of WNK1 inhibitors such as WNK 463 has been verified in vivo, which makes WNK1 kinase the emerging target for screening novel diuretics [9].

Natural products have a long history of being used as medicines to treat a variety of human diseases and are a valuable source of safe and extremely effective diuretics. The discovery and development of novel diuretic agents from natural products represent an attractive avenue [10]. The genus *Chimaphila* is a typical member of the Ericaceae family, which grows naturally in Bhutan, China, Japan, Korea, and Russia; it comprises about five species around the world, of which three species (one of which is endemic) can be found in China. *Chimaphila japonica* Miq. is a perennial herbaceous plant that has diuretic, astringent, analgesic, and other effects; and it can treat various conditions such as edema, hydrops, etc. [11]. At present, little research has been carried out on the chemical composition of the plant; the biological activity is mainly directed towards crude extracts, and the pharmacodynamic material basis is unclear. To date, only a few terpenoids, flavonoids, sterols, quinoids, and phenolic glycosides have been reported [12,13]. Therefore, an in-depth study of the active ingredients of *C. japonica* is essential. In our continuing search for potent diuretic agents from medicinal plants, petroleum ether (PE), ethyl acetate (EtOAc), and *n*-butyl alcohol (*n*-BuOH) soluble fractions from the 75% ethanol extract of whole-plant *C. japonica* were evaluated. Herein, the diuretic bioguided isolation of the active *n*-BuOH constituents of the soluble fraction, together with the diuretic activity of some of the isolated compounds, is evaluated and the possible diuretic mechanisms of the active compounds are investigated.

## 2. Results and Discussion

### 2.1. Structure Elucidation

**Figure 1:**
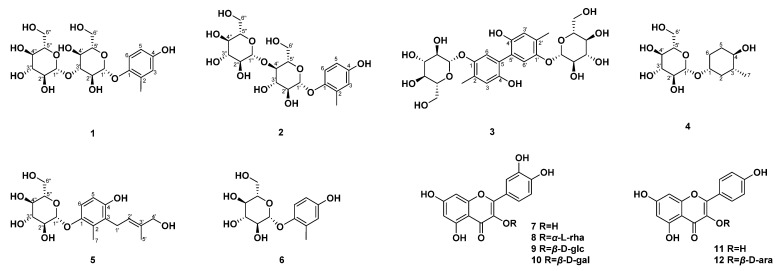
Structures of isolated compounds **1**–**12**.

The 75% ethanol extract from the whole-plant *C. japonica* was partitioned into three fractions via liquid–liquid partition using PE, EtOAc, and *n*-BuOH. The crude extracts and fractions were tested for cytotoxicity in vitro. The results show that the ethanol extract and EtOAc and *n*-BuOH fractions showed no or minor toxicity relative to MDCK cells, and could be used for subsequent activity testing. In vitro assays were performed on the crude extract and selected fractions against a new type of cell screening model for diuretic agents, in which the transport of Na^+^ and Cl^−^ is an essential indicator for the study of diuretic activity. Compared with the ethanol extract (9.23%) and the EtOAc fractions (10.11%), the *n*-BuOH fraction showed better inhibitory activity on Na^+^ transport, with an inhibition rate of 15.40%. The effect of these fractions on the suppression of Cl^−^ transport is not obvious. The *n*-BuOH fraction was further chemically investigated to obtain 12 compounds including three previously unreported phenols: 3′-*O*-*β*-d-glucopyranosyl-isohomoarbutin (**1**), 4′-*O*-*β*-d-glucopyranosyl-isohomoarbutin (**2**), and 5-5′-dehydro-di (2-methyl-4-hydroxy-phenyl-1-*O*-*β*-d-glucopyranoside) (**3**); one new cyclohexanol: (1*R*,3*R*,4*R*)-3-methylcyclohexanol *β*-d-glucopyranoside (**4**); and one first named new phenol: 3-[(*E*)-4-hydroxy-3-methyl-2-butenyl]-4-hydroxy-2-methylphenyl-*O*-*β*-d-glucopyranoside (**5**) [14]; together with: isohomoarbutin (**6**) [15], quercetin (**7**) [16], quercitrin (**8**) [17], isoquercitrin (**9**) [17], hyperoside (**10**) [18], kaempferol (**11**) [19], and juglanin (**12**) [20] (Figure 1).

**Figure 2:**
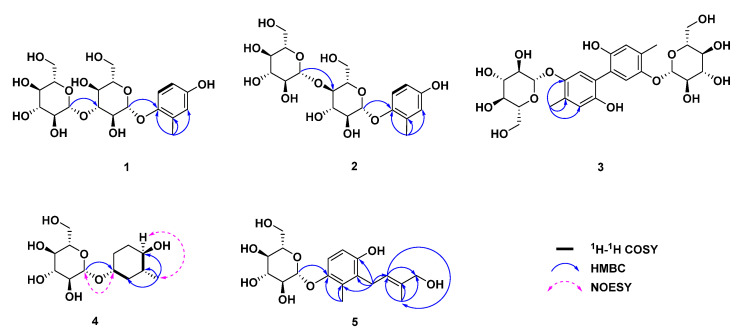
Key ^1^H–^1^H COSY, NOESY, and HMBC correlations of compounds **1**–**5**.

Compound **1** was obtained as a white amorphous powder. Its molecular formula was determined as C_19_H_28_O_12_ via HR-ESI-MS at *m*/*z* 447.1505 [M − H]^−^ (calcd. 447.1508). The ^1^H NMR spectrum of **1** (Table 1) displayed signals for three aromatic protons for an ABX spin system at *δ*_H_ 6.98 (1H, d, *J* = 8.7 Hz, H-6), 6.57 (1H, d, *J* = 2.7 Hz, H-3), and 6.52 (1H, dd, *J* = 8.6, 3.0 Hz, H-5); two anomeric protons at *δ*_H_ 4.74 (1H, d, *J* = 7.6 Hz, H-1′) and 4.59 (1H, d, *J* = 7.7 Hz, H-1″); and one methyl group at *δ*_H_ 2.21 (3H, s, H-7). The ^13^C NMR (Table 1) together with HMQC spectrums exhibited a total of 19 carbon signals, which were assigned to six aromatic carbons at *δ*_C_ 153.6 (C-4), 150.6 (C-1), 130.6 (C-2), 118.7 (C-6), 118.1 (C-3), and 113.8 (C-5); two sets of hexose groups at *δ*_C_ 105.3 (C-1″), 103.8 (C-1′), 88.0 (C-3′), 78.2 (C-3″), 77.8 (C-5′), 77.7 (C-5″), 75.5 (C-2″), 74.4 (C-2′), 71.6 (C-4″), 69.9 (C-4′), 62.6 (C-6″), and 62.5 (C-6′); and one methyl group at *δ*_C_ 16.6 (C-7). The ^1^H and ^13^C NMR spectra indicated that the skeleton of compound **1** was a phenol with two sugar moieties. According to the overall analysis, compound **1** was similar to compound 6 (isohomoarbutin) based on their similar NMR data [15], with the exception that compound **1** had one more hexose group, which resulted in the conclusion that **1** was the analogue of isohomoarbutin. After the acid hydrolysis of **1**, the two sugar residues were confirmed to be D-glucose via the HPLC assay, and the retention time was consistent with the standard substance of sugar (retention time: 20.112 min). The coupling constant (*J* = 7.6 Hz, H-1′ and *J* = 7.7 Hz, H-1″) of the anomeric protons revealed that D-glucopyranosyl units were both in the *β*-configuration. The sugar linkages were established based on the key HMBC correlations (Figure 2) from H-1″ (*δ*_H_ 4.59) to C-3′ (*δ*_C_ 88.0) and H-1′ (*δ*_H_ 4.74) to C-1 (*δ*_C_ 150.6), which indicated that the two glucopyranosyl groups were located at the C-3′ and C-1 positions, respectively. The HMBC correlations (Figure 2) between H-7 (*δ*_H_ 2.21) and C-1 (*δ*_C_ 150.6), C-2 (*δ*_C_ 130.6) and C-3 (*δ*_C_ 118.1) indicate that the methyl group was linked to C-2. Therefore, the structure of compound **1** was established as 3′-*O*-*β*-d-glucopyranosyl-isohomoarbutin.

Compound **2** was obtained as a white amorphous powder. Its molecular formula was determined as C_19_H_28_O_12_ via HR-ESI-MS at *m*/*z* 447.1507 [M − H]^−^ (calcd. 447.1508). By comparing both the NMR data (Table 1) of compounds **2** and **1**, nearly identical data indicated that **2** was a glycosylated phenol, and the only significant difference between the two compounds was the sugar moiety. The acid hydrolysis of **2** produced two D-glucose as sugar residues via the HPLC assay. The coupling constants (*J* = 7.0 Hz, H-1′ and *J* = 7.7 Hz, H-1″) of the anomeric protons revealed that two D-glucopyranosyl units were both in the *β*-configuration. The sugar linkages were established based on the key HMBC correlations (Figure 2) between H-1″ (*δ*_H_ 4.60) and C-4′ (*δ*_C_ 80.4), and between H-1′ (*δ*_H_ 4.76) and C-1 (*δ*_C_ 150.5), which indicated that the two glucopyranosyl groups were located at the C-4′ and C-1 positions. On the basis of this evidence and the spectral data, the structure of compound **2** was 4′-*O*-*β*-d-glucopyranosyl-isohomoarbutin.

Compound **3** was obtained as colorless needles. Its molecular formula was determined as C_26_H_34_O_14_ via HR-ESI-MS at *m*/*z* 569.1875 [M − H]^−^ (calcd. 569.1875). According to the ^1^H- and ^13^C-NMR spectral data (Table 2), the signals at *δ*_H_ 7.12 (1H, s, H-6) and 6.70 (1H, s, H-3) defined a 1,2,4,5-tetrasubstituted aromatic ring, which was confirmed in the ^13^C-NMR spectrum by four quaternary aromatic carbon peaks at *δ*_C_ 151.3 (C-1), 149.8 (C-4), 130.4 (C-2), and 125.4 (C-5); and two methine peaks at *δ*_C_ 120.9 (C-6) and 119.4 (C-3). The high field of the ^1^H NMR spectrum showed one aromatic methyl at *δ*_H_ 2.26 (3H, s, H-7). According to the overall analysis, compound **3** was similar to 5-5′-dehydro-di(3-methyl-4-hydroxy-phenyl-1-*O*-*β*-d-glucopyranoside) [21], an homoarbutin analogue, except that the methyl group position was different. The linkage between the methyl group and aromatic ring, established by an HMBC experiment (Figure 2), concluded that **3** was the analogue of isohomoarbutin. Further analysis of the ^13^C NMR data for a quaternary carbon at C-5 (*δ*_C_ 125.4) revealed that compound **3** should be a symmetric dehydro isohomoarbutin dimer with a linkage via the C-5/C-5′ bond. It was also supported by HR-ESI-MS data analysis. The glucosyl moiety was identified via the acid hydrolysis of **3** and comparisons with authentic sample (D-glucose). The *β*-configuration of anomeric carbon was determined via the coupling constant (*J* = 7.4 Hz) of anomeric protons. With all this evidence, compound **3** was identified as 5-5′-dehydro-di (2-methyl-4-hydroxy- phenyl-1-*O*-*β*-d-glucopyranoside).

Compound **4** was obtained as colorless oil. Its molecular formula was determined as C_13_H_24_O_7_ via HR-ESI-MS at *m*/*z* 293.1596 [M + H]^+^ (calcd. for 293.1595). The ^1^H NMR spectrum (Table 3) of **4** displayed signals for: one anomeric proton at *δ*_H_ 4.45 (1H, d, *J* = 7.8 Hz, H-1′); two oxygenated methines at *δ*_H_ 3.78 (1H, m, H-1), and 3.71 (1H, m, H-4); three methylenes at *δ*_H_ 1.77, 1.51 (2H, m, H-2), 1.91, 1.61 (2H, m, H-5), and 1.90, 1.55 (2H, m, H-6); one methine proton at *δ*_H_ 1.64 (1H, m, H-3); and one methyl group at *δ*_H_ 1.03 (3H, d, *J* = 6.7 Hz, H-7). The ^13^C NMR (Table 3) and DEPT 135 and HMQC spectrums (Figure 2) exhibited a total of 13 carbon signals. These included: one hexose at *δ*_C_102.3 (C-1′), 78.0 (C-5′), 77.9 (C-3′), 75.1 (C-2′), 71.7 (C-4′), and 62.8 (C-6′); two olefinic carbons at *δ*_C_78.4 (C-1) and 69.8 (C-4); one methine carbon at *δ*_C_ 36.5 (C-3); three methylene carbons at *δ*_C_ 36.5 (C-2), 32.3 (C-6), and 26.3 (C-5); and one methyl group at *δ*_C_ 18.7 (C-7). The ^1^H-^1^H COSY correlations (Figure 2) were observed relative to H-7 (*δ*_H_ 1.03), H-3 (*δ*_H_ 1.64), and H-4 (*δ*_H_ 3.71), and the HMBC correlations were observed from H-7 (*δ*_H_ 1.03) to C-2 (*δ*_C_ 36.5), C-3 (*δ*_C_ 36.5), and C-4 (*δ*_C_ 69.8), which indicated that the methyl group was located at the C-3 position. According to the overall analysis, compound **4** was similar to (1*R*,2*R*)-2-methylcyclohexanol [22], except for having an extra hexose group at C-1 position in **4**. After the acid hydrolysis of **4**, the sugar residue was confirmed to be D-glucose by the HPLC assay. The coupling constant (*J* = 7.8 Hz, H-1′) of the anomeric protons revealed that D-glucopyranosyl unit comprised the *β*-configuration. The HMBC correlations (Figure 2) from H-1′ (*δ*_H_ 4.45) to C-1 (*δ*_C_ 150.5) indicated that the glucopyranosyl group was linked to C-1. According to the report [23], the configuration of C-1 could be identified based on the chemical shift of the sugar’s anomeric carbon. Anomeric carbon occurs around *δ*_C_ 103.5 where it comprises the *R* configuration, while it comprises the *S* configuration at around *δ*_C_ 106.3. The anomeric carbon at *δ*_C_ 102.3 (C-1′) suggested that the configuration of C-1 in **4** was *R*. The NOESY correlations between H-4 (*δ*_H_ 3.71) and H-7 (*δ*_H_ 1.03) were detected, and this suggested that these protons were co-facial positions. In contrast, the absence of correlations between H-4 (*δ*_H_ 3.71)/H-7 (*δ*_H_ 1.03) and H-1 (*δ*_H_ 3.78) indicated that H-1 (*δ*_H_ 3.78) comprised a different orientation. Therefore, the structure of compound **4** was established as (1*R*,3*R*,4*R*)*-*3- methylcyclohexanol *β*-d-glucopyranoside.

Compound **5** was isolated as a white amorphous powder. The ^1^H NMR spectrum (Table 4) of **5** displayed signals for a pair of AB-type aromatic protons at *δ*_H_ 6.87 (1H, d, *J* = 8.8 Hz, H-6) and 6.55 (1H, d, *J* = 8.8 Hz, H-5); a *cis*-coupled olefinic proton at *δ*_H_ 5.33 (1H, dd, *J* = 6.8, 5.6 Hz, H-2′); oxygenated methylene at *δ*_H_ 3.89 (2H, s, H-4′); a methylene group at *δ*_H_ 3.40 (each 2H, m, H-1′); two methyl groups at *δ*_H_ 2.20 (3H, s, H-7) and 1.80 (3H, s, H-5′); a set of signals assignable to one hexose group; an anomeric proton signal at *δ*_H_ 4.66 (1H, d, *J* = 7.4 Hz, H-1″); and six other proton signals at *δ*_H_ 3.86–3.32. Consistent with the ^1^H NMR spectral analysis, the ^13^C NMR spectrum of **5** (Table 4) also revealed the presence of: an aromatic ring at *δ*_C_ 151.5 (C-4), 150.6 (C-1), 129.1 (C-2), 128.3 (C-3), 116.2 (C-6), and 113.3 (C-5); two olefinic carbons at *δ*c 135.3 (C-3′) and 125.4 (C-2′); oxygenated methylene at *δ*c 69.0 (C-4′); methylene at *δ*c 26.2 (C-1′); two methyls at *δ*c 14.0 (C-5′) and 12.6 (C-7); and one hexose, including an anomeric carbon at *δ*_C_ 104.3 (C-1″) and five other carbons at *δ*c 78.2–62.6. The acid hydrolysis of **5** produced D-glucose, which was determined to have a *β*-configuration based on the large coupling constants of the anomeric proton [*δ*_H_ 4.72 (d, *J* = 7.6 Hz)].

The connections of the functional groups were determined mainly by the HMBC spectrum. In the HMBC spectrum (Figure 2), correlations between H-2′/C-4′, C-5′; H-5′/C-2′, C-3′, and C-4′; and H-1′/C-2′ were observed, indicating the presence of a 4-hydroxy-3-methyl-2-butenyl moiety. This group was located at C-3, as deduced from the cross peaks between the methylene protons H-1′ (*δ*_H_ 3.40) and C-4 (*δ*c 151.5), C-2 (*δ*c 129.1), and C-3 (*δ*c 128.3) separately. The glucose moiety located at C-1 was evidenced by correlations between the proton signal at *δ*_H_ 4.66 (H-1″) and the carbon signal at *δ*c 150.6 (C-1). The proton signal at *δ*_H_ 2.20 (H-7) correlated with the carbon signal at *δ*c 150.6 (C-1), *δ*c 129.1 (C-2), and *δ*c 128.3 (C-3), indicating that the methyl group was located at C-2. Therefore, in a comparison of NMR data with the literature, the structure of compound **5** was established as 3-[(*E*)-4-hydroxy-3-methyl-2-butenyl]-4-hydroxy-2- methylphenyl-*O*-*β-*d*-*glucopyranoside [14].

### 2.2. In Vitro Cytotoxicity

First, we used the MTT method to examine the cytotoxicity of the compounds (**1**–**12**) on MDCK cells at a concentration of 100 µmol/L, and the results are shown in Table 5. Based on the experimental results, all compounds, with the exception of **2** and **3,** showed no or minor toxicity relative to MDCK cells and could be used for subsequent activity testing.

### 2.3. In Vitro Diuretic Activity 

Generally, the transport of Na^+^ and Cl^−^ plays an essential role in glomerular filtration and tubular reabsorption. In this study, a Transwell chamber seeded with MDCK cells was used to simulate the renal tubules and investigate the inhibitory effect of compounds (**1**, **4**–**12**) on NaCl transport in the renal tubules at 100 µmol/L. As shown in Table 6, all compounds, except compound **10**, exhibited highly inhibitory activity on Na^+^ transport (*p* < 0.0001) compared to the blank group. As for the transport of Cl^−^, compounds **1**, **4**, **6**–**7**, and **9**–**11** exhibited extremely inhibitory activity (*p* < 0.0001), compound **5** exhibited good inhibitory activity (*p* < 0.001), and compound **12** has a general inhibitory activity (*p* < 0.5). The results showed that some compounds exhibited excellent inhibitory activity on Na^+^ transport, particularly **4**, **7**, and **11**, with inhibition rates higher than 20%. On the other hand, compounds **7** and **11** exhibited a strong inhibitory effect on Cl^−^ transport (20.72% and 27.68%), which was significantly higher than or close to the positive control hydrochlorothiazide (23.42%).

**Figure 3:**
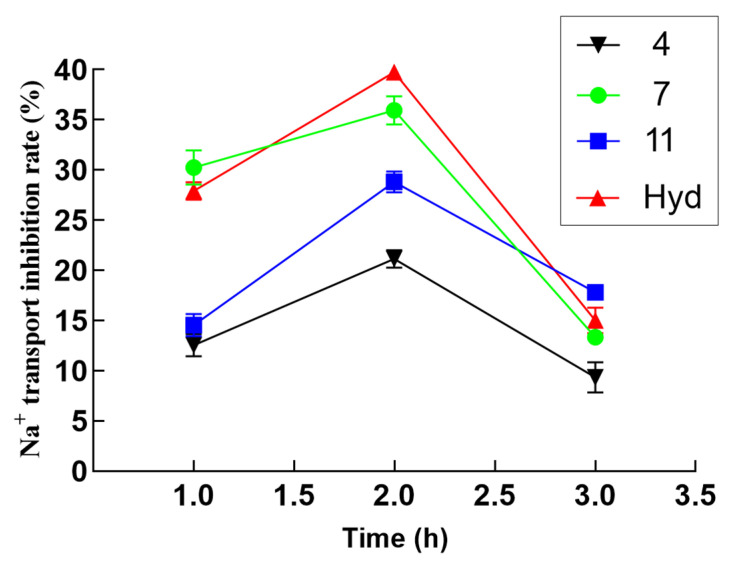
Na^+^ transport inhibition activity of compounds **4**, **7**, and **11** at 1, 2, and 3 h.

**Figure 4:**
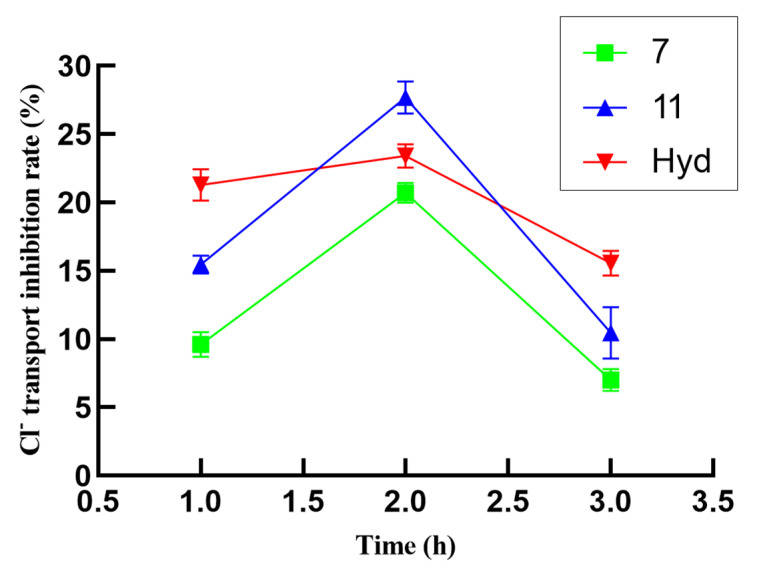
Cl^−^ transport inhibition activity of compounds **7** and **11** at 1, 2, and 3 h.

In order to explore the relationship between the Na^+^ and Cl^−^ transport inhibition activity of potential compounds and time, the inhibitory activities of compounds with inhibition rates that were higher than 20% were further evaluated at 1, 2, and 3 h. It can be observed in the data shown in Figure 3 that the transport inhibition rates of these compounds reached a peak (greater than 20%) during the second hour. The inhibition rate of compound **7** on Na^+^ transport inhibition was more than 30.24% at 1 h, which was better than that of positive control hydrochlorothiazide (27.91%). The suppression rate of Na^+^ transport inhibitory activity in compound **11** exceeds that of hydrochlorothiazide at 3 h, and the suppression rate may be more stable. As shown in Figure 4, the inhibition rates of compounds **7** and **11** on Cl^−^ transport reached a peak (greater than 20%) during the second hour, and the inhibitory rate of compound **11** for Cl^−^ transport inhibition activity exceeded 27.68%, which exceeded the positive control (hydrochlorothiazide, 23.42%). Unfortunately, the stability of these compounds is relatively weak. In short, the preliminary test results demonstrated that compounds **4**, **7**, and **11** have potential for application in diuretic activity.

### 2.4. Molecular Docking

Based on the results obtained in previous in vitro experiments, compounds **4**, **7**, and **11**, which have better inhibitory activity on Na^+^ and Cl^−^ transports, were selected for molecular docking studies to further explore the diuretic mechanisms of the selected compounds.

**Figure 5:**
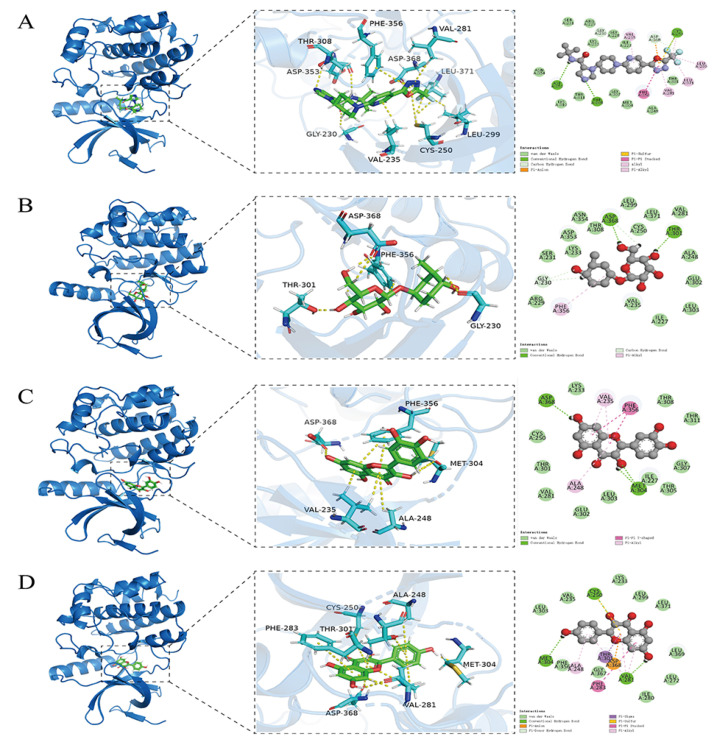
Interaction modes of WNK463 (**A**), compounds **4** (**B**), **7** (**C**), and **11** (**D**) with WNK1 kinase domain (**A**).

WNK1 kinase was employed to evaluate the diuretic effects of selected compounds by docking them into the active site of the WNK1 kinase domain (PDB ID 5DRB) [24]. WNK463 was re-docked to the active site to validate docking reliability. The results indicated the binding mode of co-crystallized and re-docked WNK463 was almost the same in the active site of the WNK1 kinase domain in Figure 5A (binding energy: −7.47 kcal/mol).

Compared with WNK463, compound **4** mainly formed two hydrogen bonds with Asp368 and Thr301, which were key for Na^+^ and Cl^−^ transport. In addition, the hydrophobic interaction with Phe356 was essential for the binding of compound **4** and the WNK1 kinase domain, with a binding energy of −6.12 kcal/mol (Figure 5B). In this study, compound **7** formed two hydrogen bonds with Asp368 and Met304, and hydrophobic interactions with Val235, Ala248, and Phe356, in Figure 5C (binding energy: −5.87 kcal/mol). Detailed interaction analyses of compound **11** revealed hydrogen bond interactions with Met304, Val281, and Cys250, and hydrophobic interactions with the Ala248, Thr301, and Val281 of WNK1, with a binding energy of −6.34 kcal/mol (Figure 5D).

It is commonly believed that the lower the binding energy, the stronger the binding force of the two molecules. In addition, if the binding energy is below −5 kcal/mol, the two molecules are considered to be strongly bound [25]. Molecular docking results showed that the selected compounds have a certain binding capacity. Thus, it can be observed that the diuresis potential of the active compounds can be realized by inhibiting the activity of the WNK1 kinase domain. Their binding driving forces comprise hydrophobic and hydrogen bond interactions.

## 3. Materials and Methods

### 3.1. Chemistry 

The NMR spectra were obtained using a Bruker AV 300 MHz spectrometer (Burker, Fallanden, Switzerland) in CD_3_OD and TMS (tetramethylsilane) as the internal standard. High-resolution electrospray ionization mass spectra (HR-ESIMS) were recorded using a UHPLC–Q Exactive Orbitrap–MS (Thermo Fisher Scientific, Boston, MA, USA). Optical rotations were measured with a Rudolph Autopol I automatic polarimeter. Column chromatography was performed using normal-phase silica gel (200–300 mesh, Branch of Qingdao Haiyang Chemical Co., Ltd., Qingdao, China). Thin-layer chromatography (TLC) was conducted on pre-coated silica gel GF_254_ glass plates (200 × 200 mm, Qingdao Haiyang Chemical Co., Ltd., Qingdao, China) and RP-18 F_254_S (Merck KGaA, Darmstadt, Germany). All reagents and solvents were of reagent grade or purified according to standard methods before use. 

### 3.2. Plant Material and Identification 

The *C. japonica* was harvested from the Changbai Mountain area, Jilin Province, China, in July 2018, and it was identified by Prof. Ming-shan Zheng (School of Pharmaceutical Sciences, Yanbian University, China). A voucher specimen (20180705-XDC) was deposited at the Department of Pharmacognosy, School of Pharmaceutical Sciences, Yanbian University, China.

### 3.3. Extraction and Isolation

The air-dried whole plants of *C. japonica* (3.3 kg) were extracted with 75% ethanol (3 × 40 L) via reflux. The extract was freed from the solvent using a rotavapor to yield 642.7 g of EtOH extract. Part of the crude extract (627.2 g) was suspended with distilled water and successively partitioned into PE (3 × 1.4 L), EtOAc (3 × 1.4 L), and *n*-BuOH (3 × 1.4 L) sequentially to obtain PE, EtOAc, *n*-BuOH, and aqueous fractions.

The *n*-BuOH fraction (160.2 g) was subjected to silica gel column chromatography (CC) with CH_2_Cl_2_–MeOH–H_2_O (40:1:0–3:1:0.1, *v*/*v*/*v*) to afford eight fractions (Fr. 1–8). Fr. 2 (12.0 g) was subjected to silica gel CC using CH_2_Cl_2_–MeOH (30:1–0:100, *v*/*v*) as the mobile phase to give eight subfractions (Fr. 2-1–Fr. 2-8). Fr. 2-2 was separated successively using a Sephadex LH-20 column by eluting with CH_2_Cl_2_–MeOH (3:7, *v*/*v*) and a reverse-phase (RP) column by eluting with MeOH–H_2_O (30:1–1:0, *v*/*v*) to give compounds **7** (5.0 mg), **8** (7.0 mg), and **9** (7.0 mg). Fr. 2-5 was subjected to RP CC by eluting with MeOH–H_2_O (4:6–1:0, *v*/*v*) to yield compounds **10** (11.1 mg), **11** (17.5 mg), and **12** (8.2 mg), successively. Fr. 3 (13.0 g) was subjected to silica gel CC using CH_2_Cl_2_–MeOH (30:1–0:100, *v*/*v*) as the mobile phase to give six subfractions (Fr. 3-1–Fr. 3-6). Fr. 3-4 was subjected to RP CC by gradient elution with MeOH–H_2_O (4:6–1:0, *v*/*v*) to yield compounds **6** (118.1 mg). Fr. 5 (18.6 g) was fractionated using silica gel CC by gradient elution with CH_2_Cl_2_–MeOH (20:1–3:1, *v*/*v*) to give five subfractions (Fr. 5-1–Fr. 5-5). Fr. 5-3 was purified using a Sephadex LH-20 column by eluting with 100% MeOH to afford compound **1** (12.9 mg) and compound **2** (15.3 mg). Fr. 5-4 (3.9 g) was subjected to silica gel CC using CH_2_Cl_2_–MeOH (3:1-0:100, *v*/*v*) as the mobile phase to give seven subfractions (Fr. 5-4-1–Fr. 5-4-7). Fr. 5-4-3 was subjected to RP CC by gradient elution with MeOH–H_2_O (1:9–1:0, *v*/*v*) to yield compounds **3** (7.3 mg), **4**, (16.1 mg) and **5** (6.1 mg).

### 3.4. Characterization of the Isolates

3′-*O*-*β*-d-glucopyranosyl-isohomoarbutin (**1**): colorless needles; ${\left[\alpha \right]}_{D}^{25}$ −18.05 (*c* 0.20, MeOH); UV (MeOH) λ*_max_* (nm; log *ε*): 287 (3.46); HR-ESI-MS *m*/*z* 447.1505 [M − H]^−^ (calcd. for C_19_H_27_O_12_, 447.1508). For ^1^H and ^13^C NMR data (CD_3_OD, 300 and 75 MHz), we refer the reader to Table 1. All significant data are provided in the electronic Supplementary Materials (Figures S1–S8).

4′-*O*-*β*-d-glucopyranosyl-isohomoarbutin (**2**): colorless needles, ${\left[\alpha \right]}_{D}^{25}$ −20.11 (*c* 0.22, MeOH); UV (MeOH) λ*_max_* (nm; log *ε*): 294 (3.46); HR-ESI-MS *m*/*z* 447.1507 [M − H]^−^ (calcd. for C_19_H_27_O_12_, 447.1508). For ^1^H and ^13^C NMR data (CD_3_OD, 300 and 75 MHz), we refer the reader to Table 1. All significant data are provided in the electronic Supplementary Materials (Figures S9–S16).

5-5′-dehydro-di(2-methyl-4-hydroxy-phenyl-1-*O*-*β*-d-glucopyranoside) (**3**): colorless needles, ${\left[\alpha \right]}_{D}^{25}$ −20.36 (*c* 0.25, MeOH); UV (MeOH) λ*_max_* (nm; log *ε*): 219 (3.35), 250 (3.11), 300 (3.10); HR-ESI-MS *m*/*z* 569.1875 [M − H]^−^ (calcd. for C_26_H_33_O_14_, 569.1875). For ^1^H and ^13^C NMR data (CD_3_OD, 300 and 75 MHz), we refer the reader to Table 2. All significant data are provided in the electronic Supplementary Materials (Figures S17–S23).

(1*R*,3*R*,4*R*)-3-methylcyclohexanol *β*-d-glucopyranoside (**4**): colorless needles, ${\left[\alpha \right]}_{D}^{25}$ −32.16 (*c* 0.10, MeOH); UV (MeOH) λ*_max_* (nm; log *ε*): 287 (3.10); HR-ESI-MS *m*/*z* 293.1596 [M + H]^+^ (calcd. for C_13_H_25_O_7_, 293.1595). For ^1^H and ^13^C NMR data (CD_3_OD, 300 and 75 MHz), we refer the reader to Table 3. All significant data are provided in the electronic Supplementary Materials (Figures S24–S33).

### 3.5. In Vitro Cytotoxicity Assays 

Each compound was evaluated for cytotoxicity on DMCK cells using MTT assays. A 96-well plate containing 1 × 10^4^ cells per well was seeded with logarithmic growth-phase DMCK cells, which were then cultured for 24 h in a cell culture incubator. After that, the culture medium was changed to one that included medication (100 µmol/L for the treatment groups, 100 µmol/L for the control group, or 100 µmol/L for the positive control group). In total, 100 µL of the appropriate solution was added to each well, and they were then incubated for a further 24 h. After that, culture media were taken out and changed for a DMEM solution that included 20 µL of MTT (5 mg/mL). After incubating for 4 h, the liquid in each well was removed and replaced with 150 µL of DMSO, which was shaken for 10 min to ensure thorough mixing. The absorbance at 490 nm was measured to determine the optical density (OD) of each well. The cell growth inhibition rate was calculated as follows: cell growth inhibition rate (%) = (OD_Blank_ − OD_Experimental_)/OD_Blank_ × 100%. 

### 3.6. In Vitro Diuretic Activity Assay 

Log-phase MDCK cells were seeded in the upper chambers of Transwell plates (4 × 10^4^ cells/well), and 800 µL of complete culture medium was added to the lower chambers. After 24 h of incubation at 37 ℃, the electrical resistance of the upper chamber cells was measured one by one (R = (R_cell_ − R_blank_) × 0.04π). When the electrical resistance of the upper chamber cells reached ≥ 300 Ω cm^2^, the upper chamber medium was removed and replaced with the drug solution. The blank group (given normal saline), experimental group (**1**, **4**–**12**, 100 µmol/L), and hydrochlorothiazide group (200 µmol/L) were set, and each well was treated with 200 µL of the corresponding solution. The cells were then further incubated for 24 h. After that, the upper and lower chamber fluids were removed, and 200 µL of NaCl solution (21 mg/mL) was added to the upper chamber, while 800 µL of DMEM was added to the lower chamber for continued incubation. At 1, 2, and 3 h, 50 µL of the lower chamber fluid was taken, and the OD values were measured using Na^+^ and Cl^−^ detection kits. The transport inhibition rate (%) was calculated as follows: transport inhibition rate (%) = (OD_blank_ − OD_experimental_)/OD_blank_ × 100%.

### 3.7. Molecular Docking Study 

To explore the interaction between different compounds and the WNK1 kinase, the crystal structure of the WNK1 kinase domain in complex with WNK463 (PDB ID 5DRB) was selected to perform molecular docking studies [24]. Using the PubChem database (http://pubchem.ncbi.nlm.nih.gov/, accessed on 5 January 2024) for the 2D structure of small molecule ligands, the 2D structures were fed into Chem Office 2022 software to produce their 3D structures. Then, the RCSB PDB database (http://www.rcsb.org/, accessed on 5 January 2024) was used to screen the protein targets, and the crystal structure with high resolution was used as the molecular docking receptor. The PyMOLWin 2.6 software was used to dewater and dephosphate the protein. The Molecular Operating Environment 2019 software was used to minimize the energy of the compounds, pretreat the target proteins, and find the active pockets. Finally, MOE 2019 was run for molecular docking. The results were visualized using PyMOL and Discovery Studio 2019 software.

### 3.8. Statistical Analyses 

Statistical analyses were performed by Graphpad Prism 6.0. Data were expressed as mean ± standard deviation (SD) based on at least three independent experiments. And differences between groups were analyzed by one-way analysis of variance and Student’s *t*-test. A *p* < 0.05 was considered to be statistically significant, *p* < 0.01 was considered as a significant difference, *p* < 0.0001 considered as an extremely significant differences.

## 4. Conclusions 

In summary, to identify compounds with potent diuretic activity, the components of *C. japonica* were separated, resulting in the identification of 12 compounds (**1**–**12**), including three previously undescribed phenols (**1**–**3**) and one new cyclohexanol (**4**). Bioassays demonstrated that compounds **4**, **7**, and **11** possess potent diuretic activity. The docking study further revealed that the diuresis potential of the active compounds could be realized by the WNK1 kinase domain. Their binding driving forces comprise hydrophobic and hydrogen bond interactions. However, in order to improve the bioavailability of active compounds, further structural modification and mechanism analysis are necessary in the future. 

## Figures and Tables

**Table 1:** ^1^H (300 MHz) and ^13^C (75 MHz) NMR data of compound **1**–**2** in CD_3_OD (*δ* in ppm).

Position	1		2	
	*δ*_H_, Mult (*J* in Hz)	*δ*_C_	*δ*_H_, Mult (*J* in Hz)	*δ*_C_
1	-	150.6	-	150.5
2	-	130.6	-	130.7
3	6.57 (d, 2.7)	118.1	6.57 (d, 2.8)	118.2
4	-	153.6	-	153.6
5	6.52 (dd, 8.6, 3.0)	113.8	6.52 (dd, 8.6, 2.9)	113.8
6	6.98 (d, 8.7)	118.7	6.96 (d, 8.7)	118.7
7	2.21 (s)	16.6	2.21 (s)	16.7
1′	4.74 (d, 7.6)	103.8	4.76 (d, 7.0)	103.8
2′	3.60 (m)	74.4	3.22 (m)	74.9
3′	3.49 (m)	88.0	3.62 (m)	76.5
4′	3.49 (m)	69.9	3.64 (m)	80.4
5′	3.32 (m)	77.8	3.64 (m)	76.5
6′	3.88 (d, 12.0) 3.71 (dd, 12.0, 5.2)	62.5	3.89 (dd, 11.2, 3.2) 3.73 (dd, 12.0, 5.2)	61.7
1″	4.59 (d, 7.7)	105.3	4.60 (d, 7.7)	104.6
2″	3.30 (1H, m)	75.5	3.54 (m)	74.8
3″	3.34 (1H, m)	78.2	3.35 (m)	78.1
4″	3.44 (1H, m)	71.6	3.35 (m)	71.4
5″	3.34 (1H, m)	77.7	3.35 (m)	77.8
6″	3.88 (d, 12.0) 3.71 (dd, 12.0, 5.2)	62.6	3.89 (dd, 11.2, 3.2) 3.73 (dd, 12.0, 5.2)	62.4

**Table 2:** This ^1^H (300 MHz) and ^13^C (75 MHz) NMR data of compound **3** in CD_3_OD (*δ* in ppm).

Position	*δ*_H_, Mult (*J* in Hz)	*δ*_C_	Position	*δ*_H_, Mult (*J* in Hz)	*δ*_C_
1 (1′)	-	151.3	Glc-1 (1′)	4.80 (d, 7.4)	104.4
2 (2′)	-	130.4	Glc-2 (2′)	3.44 (m)	75.1
3 (3′)	6.70 (s)	119.4	Glc-3 (3′)	3.39 (m)	78.1
4 (4′)	-	149.8	Glc-4 (4′)	3.37 (m)	71.6
5 (5′)	-	125.4	Glc-5 (5′)	3.39 (m)	78.0
6 (6′)	7.12 (s)	120.9	Glc-6 (6′)	3.86 (d, 11.8) 3.66 (d, 10.5)	62.6
7 (7′)	2.26 (s)	16.4			

**Table 3:** ^1^H (300 MHz) and ^13^C (75 MHz) NMR data of compound **4** in CD_3_OD (*δ* in ppm).

Position	*δ*_H_, Mult (*J* in Hz)	*δ*_C_	Position	*δ*_H_, Mult (*J* in Hz)	*δ*_C_
1	3.78 (m)	78.4	1′	4.45 (d, 7.8)	102.3
2	1.77 (d, 11.7) 1.51 (m)	36.5	2′	3.60 (m)	75.1
3	1.64 (m)	36.5	3′	3.39 (m)	77.9
4	3.71 (m)	69.8	4′	3.37 (m)	71.7
5	1.91 (d, 8.5) 1.61 (m)	26.3	5′	3.39 (m)	78.0
6	1.90 (m), 1.55 (m)	32.3	6′	3.86 (d, 11.8) 3.66 (d, 10.5)	62.8
7	1.03 (d, 6.7)	18.7			

**Table 4:** ^1^H (300 MHz) and ^13^C (75 MHz) NMR data of compound **5** in CD_3_OD (*δ* in ppm).

Position	*δ*_H_, Mult (*J* in Hz)	*δ*_C_	Position	*δ*_H_, Mult (*J* in Hz)	*δ*_C_
1	-	150.6	3′	-	135.3
2	-	129.1	4′	3.89 (s)	69.0
3	-	128.3	5′	1.80 (s)	14.0
4	-	151.5	1″	4.66 (d, 7.4)	104.3
5	6.55 (d, 8.8)	113.3	2″	3.41 (m)	75.1
6	6.87 (d, 8.8)	116.2	3″	3.30 (m)	77.9
7	2.20 (s)	12.6	4″	3.38 (m)	71.5
1′	3.40 (m)	26.2	5″	3.40 (m)	78.2
2′	5.33 (dd, 6.8, 5.6)	125.4	6″	3.68 (dd, 12.0, 5.1) 3.89 (m)	62.6

**Table 5:** Cytotoxicity of compounds **1**–**12** on MDCK Cells at 100 µmol/L.

Compound	Growth Inhibition Rate (%) ^a^	Compound	Growth Inhibition Rate (%) ^a^
**1**	4.89 ± 0.05	**7**	/
**2**	29.48 ± 0.12	**8**	/
**3**	23.31 ± 0.13	**9**	/
**4**	4.89 ± 0.05	**10**	/
**5**	4.08 ± 0.03	**11**	/
**6**	/ ^b^	**12**	/
**Hyd ^c^**	/		

^a^ Values are the mean ± SD of three replicates. ^b^ No inhibition action. ^c^ Hyd represents hydrochlorothiazide.

**Table 6:** Na^+^ and Cl^−^ transport inhibition activity of compounds **1**, **4**–**12** at 100 µmol/L.

Compound	Transport Inhibition Rate (%) ^a^		Compound	Transport Inhibition Rate (%) ^a^	
	Na^+^	Cl^−^		Na^+^	Cl^−^
**1**	−11.70 ± 1.14 ^e^	9.44 ± 0.78 ^e^	**8**	18.52 ± 1.02 ^e^	0.65 ± 1.45
**4**	21.13 ± 0.89 ^e^	12.23 ± 0.18 ^e^	**9**	6.22 ± 0.53 ^e^	−8.36 ± 2.60 ^e^
**5**	7.61 ± 0.71 ^e^	4.57 ± 0.65 ^d^	**10**	1.82 ±0.81	−13.16 ± 1.89 ^e^
**6**	10.22 ± 1.24 ^e^	12.17 ± 1.33 ^e^	**11**	28.81± 1.04 ^e^	27.68 ± 1.18 ^e^
**7**	35.95 ± 1.42 ^e^	20.72 ± 0.74 ^e^	**12**	14.18 ± 0.69 ^e^	3.27 ± 0.31 ^c^
**Hyd ^b^**	39.74 ± 0.64 ^e^	23.42 ± 0.87 ^e^			

^a^ Values are the mean ± SD of three replicates. ^b^ Hyd (hydrochlorothiazide) was used as the positive control. ^c^ * *p* < 0.05 vs. blank group. ^d^ ** *p* < 0.01 vs. blank group. ^e^ **** *p* < 0.0001 vs. blank group.

## Data Availability

Data are included within the manuscript or the Supplementary Materials.

## Supplementary Materials

The following supporting information can be downloaded at: https://www.mdpi.com/article/10.3390/molecules29051092/s1, Figure S1 ^1^H NMR spectrum of compound **1** (300 MHz, methanol-*d*_4_). Figure S2. ^13^C NMR spectrum of compound **1** (75 MHz, methanol-*d*_4_). Figure S3. ^1^H-^13^C HMQC spectrum of compound **1** (methanol-*d*_4_). Figure S4. ^1^H-^13^C HMBC spectrum of compound **1** (methanol-*d*_4_). Figure S5. ^1^H-^1^H COSY spectrum of compound **1** (methanol-*d*_4_). Figure S6. UV spectrum of compound **1**. Figure S7. HRESIMS spectrum of compound **1**. Figure S8. The HPLC spectrum of the compound **1** of D-glucopyranose. Figure S9. ^1^H NMR spectrum of compound **2** (300 MHz, methanol-*d*_4_). Figure S10. ^13^C NMR spectrum of compound **2** (75 MHz, methanol-*d*_4_). Figure S11. ^1^H-^13^C HMQC spectrum of compound **2** (methanol-*d*_4_). Figure S12. ^1^H-^13^C HMBC spectrum of compound **2** (methanol-*d*_4_). Figure S13. ^1^H-^1^H COSY spectrum of compound **2** (methanol-*d*_4_). Figure S14. UV spectrum of compound **2**. Figure S15. HRESIMS spectrum of compound **2**. Figure S16. The HPLC spectrum of the compound **2** of D-glucopyranose. Figure S17. ^1^H NMR spectrum of compound **3** (300 MHz, methanol-*d*_4_). Figure S18. ^13^C NMR spectrum of compound **3** (75 MHz, methanol-*d*_4_). Figure S19. ^1^H-^13^C HMQC spectrum of compound **3** (methanol-*d*_4_). Figure S20. ^1^H-^13^C HMBC spectrum of compound **3** (methanol-*d*_4_). Figure S21. UV spectrum of compound **3**. Figure S22. HRESIMS spectrum of compound **3**. Figure S23. The GC spectrum of the compound **3** of D-glucopyranose. Figure S24. ^1^H NMR spectrum of compound **4** (300 MHz, methanol-*d*_4_). Figure S25. ^13^C NMR spectrum of compound **4** (75 MHz, methanol-*d*_4_). Figure S26. DEPT spectrum of compound **4** in methanol-*d*_4_. Figure S27. ^1^H-^1^H COSY spectrum of compound **4** (methanol-*d*_4_). Figure S28. ^1^H-^13^C HMQC spectrum of compound **4** (methanol-*d*_4_). Figure S29. ^1^H-^13^C HMBC spectrum of compound **4** (methanol-*d*_4_). Figure S30. NOESY spectrum of compound **4** in methanol-*d*_4_. Figure S31. UV spectrum of compound **4**. Figure S32. HRESIMS spectrum of compound **4**. Figure S33. The HPLC spectrum of the compound **4** of D-glucopyranose. Figure S34. 1H NMR spectrum of compound **5** (300 MHz, methanol-*d*_4_). Figure S35. ^13^C NMR spectrum of compound **5** (75 MHz, methanol-*d*_4_). Figure S36. ^1^H-^1^H COSY spectrum of compound **5** (methanol-*d*_4_). Figure S37. ^1^H-^13^C HMQC spectrum of compound **5** (methanol-*d*_4_). Figure S38. ^1^H-^13^C HMBC spectrum of compound **5** (methanol-*d*_4_). Figure S39. UV spectrum of compound **5**. Figure S40. ^1^H-NMR spectrum of compound **6** (300 MHz, methanol-*d*_4_). Figure S41. ^13^C-NMR spectrum of compound **6** (75 MHz, methanol-*d*_4_). Figure S42. ^1^H-NMR spectrum of compound **7** (300 MHz, methanol-*d*_4_). Figure S43. ^13^C-NMR spectrum of compound **7** (75 MHz, methanol-*d*_4_). Figure S44. ^1^H-NMR spectrum of compound **8** (300 MHz, methanol-*d*_4_). Figure S45. ^13^C-NMR spectrum of compound **8** (75 MHz, methanol-*d*_4_). Figure S46. ^1^H-NMR spectrum of compound **9** (300 MHz, methanol-*d*_4_). Figure S47. ^13^C-NMR spectrum of compound **9** (75 MHz, methanol-*d*_4_). Figure S48. ^1^H-NMR spectrum of compound **10** (300 MHz, methanol-*d*_4_). Figure S49. ^13^C-NMR spectrum of compound **10** (75 MHz, methanol-*d*_4_). Figure S50. ^1^H-NMR spectrum of compound **11** (300 MHz, methanol-*d*_4_). Figure S51. ^13^C-NMR spectrum of compound **11** (75 MHz, methanol-*d*_4_). Figure S52. ^1^H-NMR spectrum of compound **12** (300 MHz, methanol-*d*_4_). Figure S53. ^13^C-NMR spectrum of compound **12** (75 MHz, methanol-*d*_4_).
