# Supplementary material for: Constituents of Chimaphila japonica and Their Diuretic Activity
Source: Molecules. 2024 Feb 29;29(5):1092. doi: 10.3390/molecules29051092 (PMC10934158; doi:10.3390/molecules29051092)
Supplement: Supplementary file 1 [file molecules-29-01092-s001.zip › molecules-2844110-supplementary.pdf]

Supplementary Materials

Constituents of *Chimaphila japonica* and Their Diuretic Activity

Yue Yu [1], Deri Hu [1], Jinze Liu [1], Chenghao Wu [1], Yuhong Sun [1], Mingyue Lang [1], Xuan Han [2], Dongzhou Kang [1], Jun Zhe Min [1,3], Hong Cui [4,*] and Mingshan Zheng [1,3,*]

1 School of Pharmaceutical Sciences, Yanbian University, Yanji 133000, China; 16604588858@163.com (Y.Y.); 15149910329@163.com (D.H.); yxh209644689@163.com (J.L.); 2023010925@ybu.edu.cn (C.W.); sunyuhong6012@163.com (Y.S.); 15948504041@163.com (M.L.); kangdz@ybu.edu.cn (D.K.); junzhemin23@163.com (J.M.)
2 School of Pharmaceutical Sciences, Jilin University, Changchun 130000, China; hanxuan199907@163.com
3 Key Laboratory of Natural Medicines of the Changbai Mountain, Ministry of Education, Yanbian University, Yanji 133000, China
4 Center of Medical Functional Experiment, Yanbian University College of Medicine, Yanji 133000, China
* Correspondence: cuihong@ybu.edu.cn (H.C.); zhengmingshan@ybu.edu.cn (M.Z.)

Table of Contents

Figure S1. ^{1}H NMR spectrum of compound **1** (300 MHz, methanol-d_4)

Figure S2. ^{13}C NMR spectrum of compound **1** (75 MHz, methanol-d_4)

Figure S3. ^{1}H-^{13}C HMQC spectrum of compound **1** (methanol-d_4)

Figure S4. ^{1}H-^{13}C HMBC spectrum of compound **1** (methanol-d_4)

Figure S5. ^{1}H-^{1}H COSY spectrum of compound **1** (methanol-d_4)

Figure S6. UV spectrum of compound **1**

Figure S7. HRESIMS spectrum of compound **1**

Figure S8. The HPLC pectrum of the compound **1** of D-glucopyranose

Figure S9. ^{1}H NMR spectrum of compound **2** (300 MHz, methanol-d_4)

Figure S10. ^{13}C NMR spectrum of compound **2** (75 MHz, methanol-d_4)

Figure S11. ^{1}H-^{13}C HMQC spectrum of compound **2** (methanol-d_4)

Figure S12. ^{1}H-^{13}C HMBC spectrum of compound **2** (methanol-d_4)

Figure S13. ^{1}H-^{1}H COSY spectrum of compound **2** (methanol-d_4)

Figure S14. UV spectrum of compound **2**

Figure S15. HRESIMS spectrum of compound **2**

Figure S16. The HPLC pectrum of the compound **2** of D-glucopyranose

Figure S17. ^{1}H NMR spectrum of compound **3** (300 MHz, methanol-d_4)

Figure S18. ^{13}C NMR spectrum of compound **3** (75 MHz, methanol-d_4)

Figure S19. ^{1}H-^{13}C HMQC spectrum of compound **3** (methanol-d_4)

Figure S20. ^{1}H-^{13}C HMBC spectrum of compound **3** (methanol-d_4)

Figure S21. UV spectrum of compound **3**

Figure S22. HRESIMS spectrum of compound **3**

Figure S23. The GC pectrum of the compound **3** of D-glucopyranose

Figure S24. ^{1}H NMR spectrum of compound **4** (300 MHz, methanol-d_4)

Figure S25. ^{13}C NMR spectrum of compound **4** (75 MHz, methanol-d_4)

Figure S26. DEPT spectrum of compound **4** in methanol-d_4

Figure S27. ^{1}H-^{1}H COSY spectrum of compound **4** (methanol-d_4)

Figure S28. ^{1}H-^{13}C HMQC spectrum of compound **4** (methanol-d_4)

Figure S29. ^{1}H-^{13}C HMBC spectrum of compound **4** (methanol-d_4)

Figure S30. NOESY spectrum of compound **4** in methanol-d_4

Figure S31. UV spectrum of compound **4**

Figure S32. HRESIMS spectrum of compound **4**

Figure S33. The HPLC pectrum of the compound **4** of D-glucopyranose

Figure S34. ^{1}H NMR spectrum of compound **5** (300 MHz, methanol-d_4)

Figure S35. ^{13}C NMR spectrum of compound **5** (75 MHz, methanol-d_4)

Figure S36. ^{1}H-^{1}H COSY spectrum of compound **5** (methanol-d_4)

Figure S37. ^{1}H-^{13}C HMQC spectrum of compound **5** (methanol-d_4)

Figure S38. ^{1}H-^{13}C HMBC spectrum of compound **5** (methanol-d_4)

Figure S39. UV spectrum of compound **5**

Figure S40. ^{1}H-NMR spectrum of compound **6** (300 MHz, methanol-d_4)

Figure S41. ^{13}C-NMR spectrum of compound **6** (75 MHz, methanol-d_4)

Figure S42. ^{1}H-NMR spectrum of compound **7** (300 MHz, methanol-d_4)

Figure S43. ^{13}C-NMR spectrum of compound **7** (75 MHz, methanol-d_4)

Figure S44. ^{1}H-NMR spectrum of compound **8** (300 MHz, methanol-d_4)

Figure S45. ^{13}C-NMR spectrum of compound **8** (75 MHz, methanol-d_4)

Figure S46. ^{1}H-NMR spectrum of compound **9** (300 MHz, methanol-d_4)

Figure S47. ^{13}C-NMR spectrum of compound **9** (75 MHz, methanol-d_4)

Figure S48. ^{1}H-NMR spectrum of compound **10** (300 MHz, methanol-d_4)

Figure S49. ^{13}C-NMR spectrum of compound **10** (75 MHz, methanol-d_4)

Figure S50. ^{1}H-NMR spectrum of compound **11** (300 MHz, methanol-d_4)

Figure S51. ^{13}C-NMR spectrum of compound **11** (75 MHz, methanol-d_4)

Figure S52. ^{1}H-NMR spectrum of compound **12** (300 MHz, methanol-d_4)

Figure S53. ^{13}C-NMR spectrum of compound **12** (75 MHz, methanol-d_4)

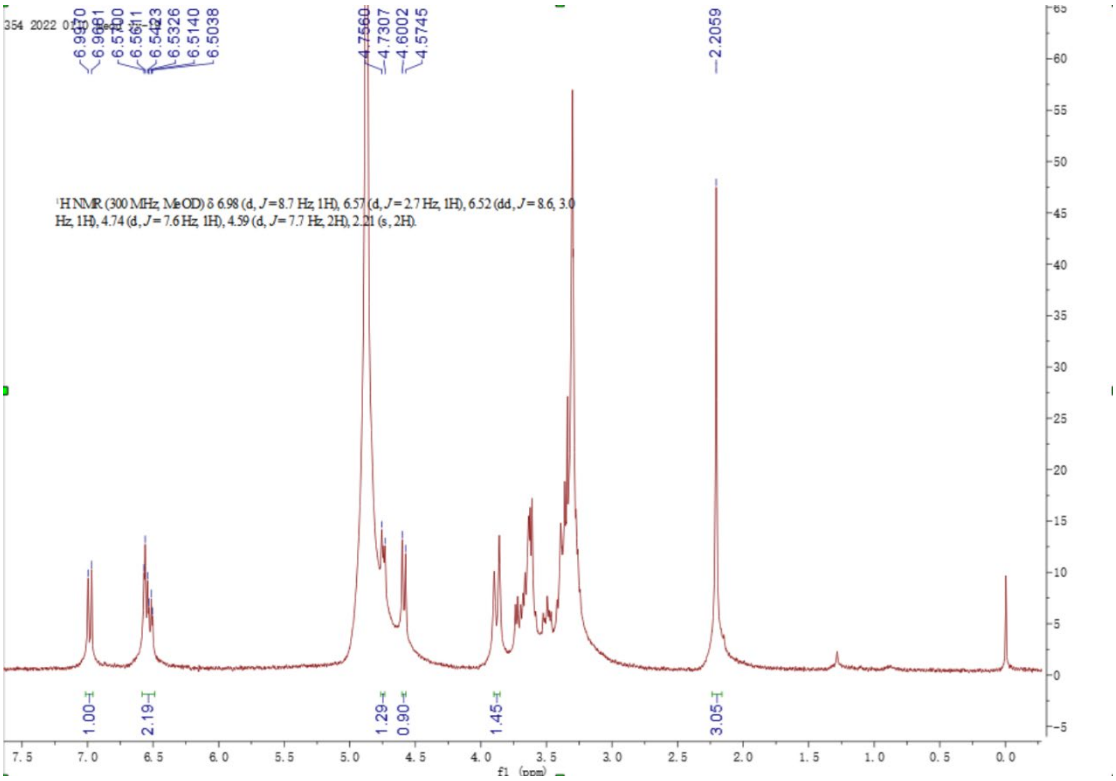

Figure S1. ^{1}H NMR spectrum of compound **1** (300 MHz, methanol-d_4)

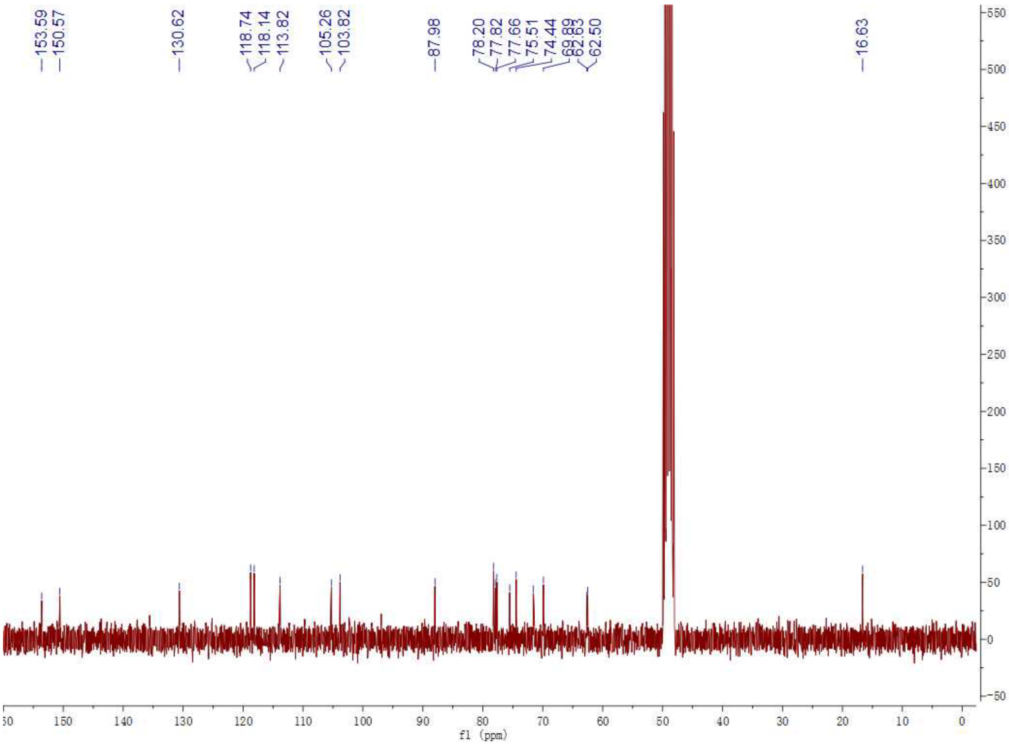

Figure S2. ^{13}C NMR spectrum of compound **1** (75 MHz, methanol-d_4)

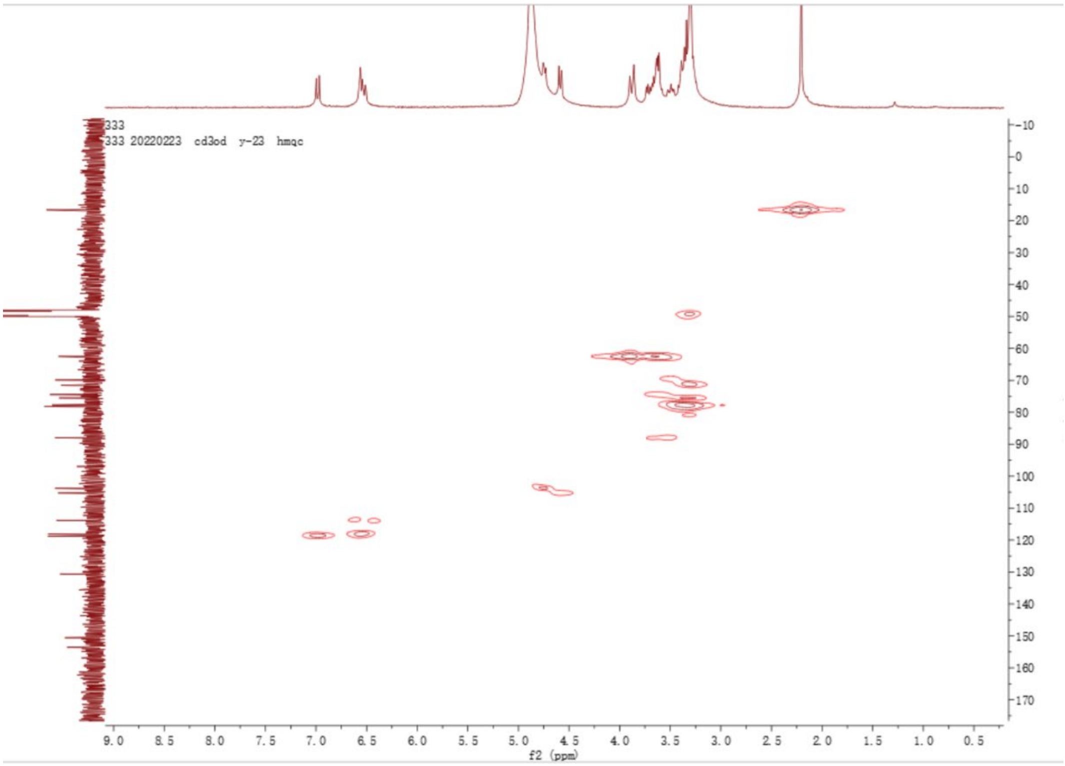

Figure S3. ^{1}H-^{13}C HMQC spectrum of compound **1** (methanol-d_4)

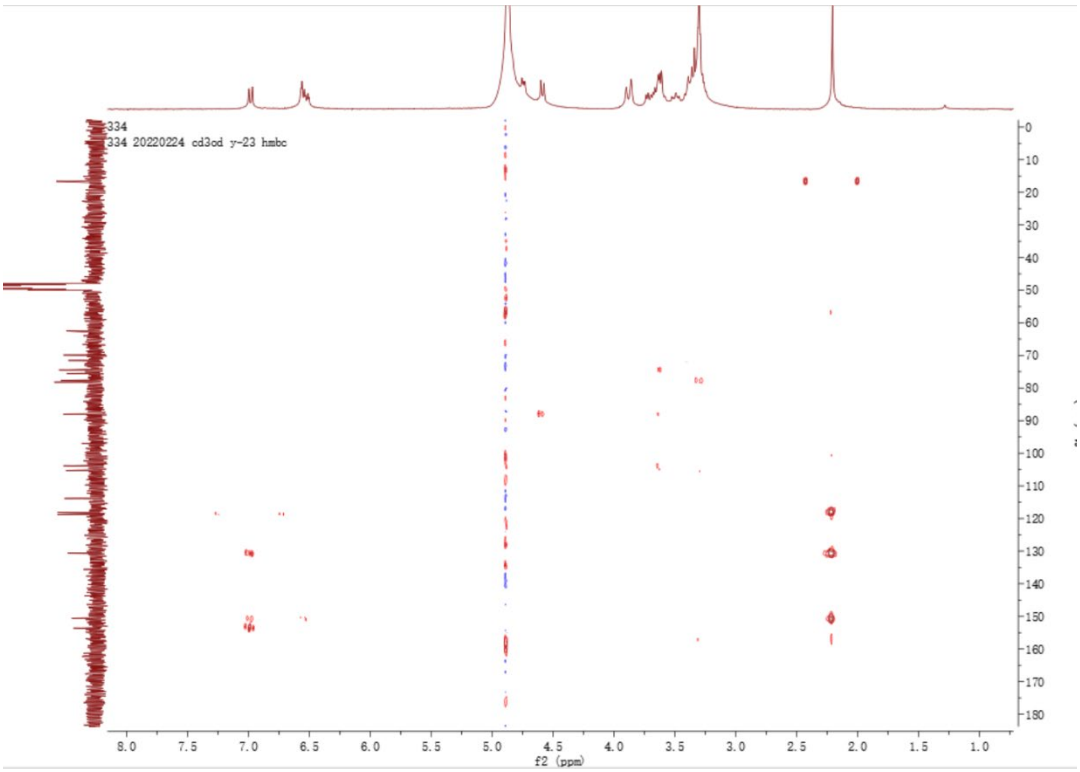

Figure S4. ^{1}H-^{13}C HMBC spectrum of compound **1** (methanol-d_4)

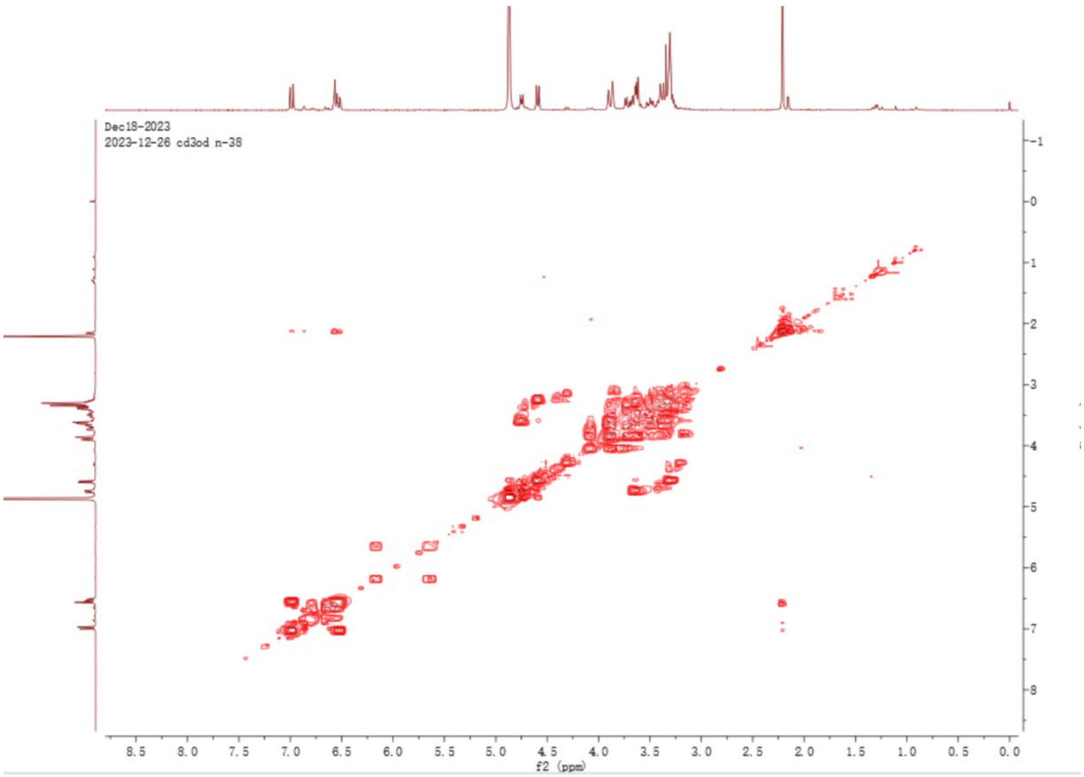

Figure S5. ^{1}H-^{1}H COSY spectrum of compound **1** (methanol-d_4)

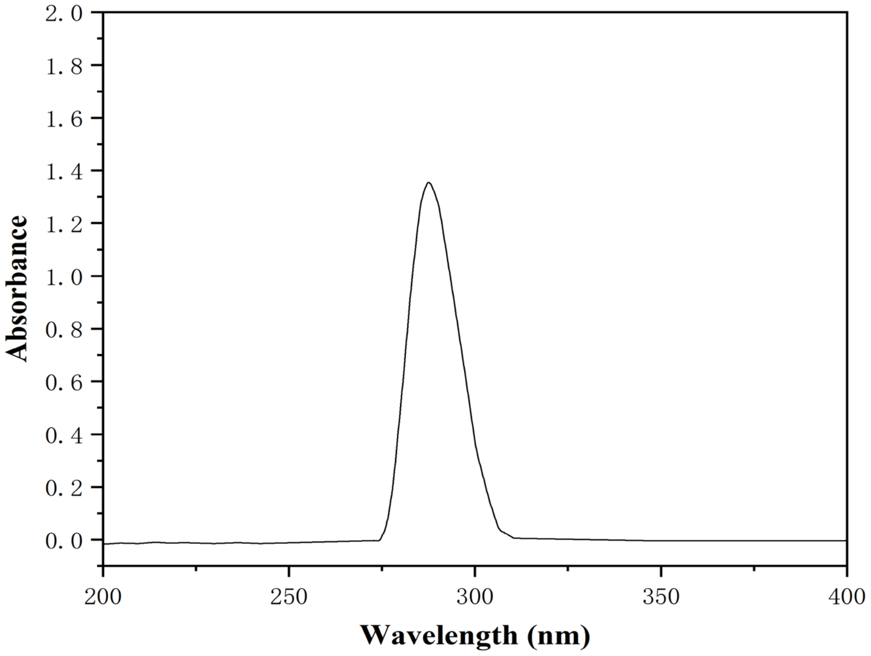

Figure S6. UV spectrum of compound **1**

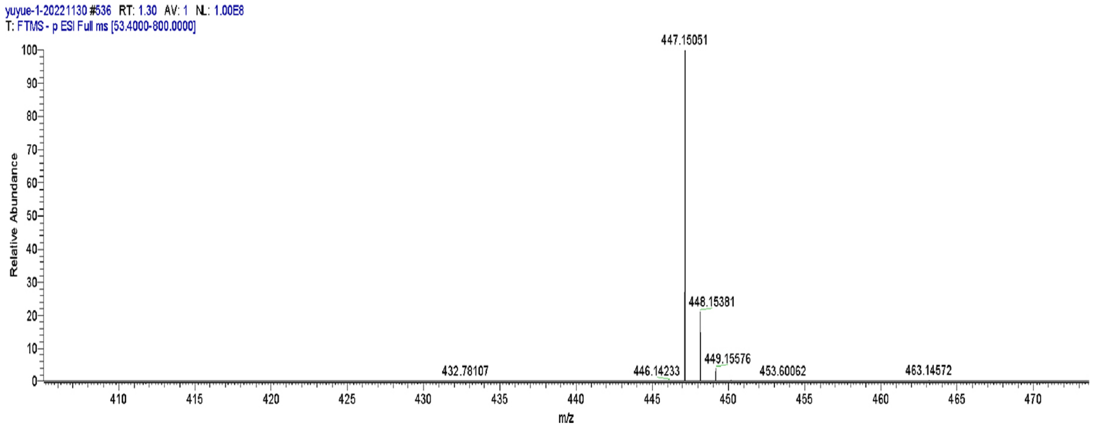

Figure S7. HRESIMS spectrum of compound **1**

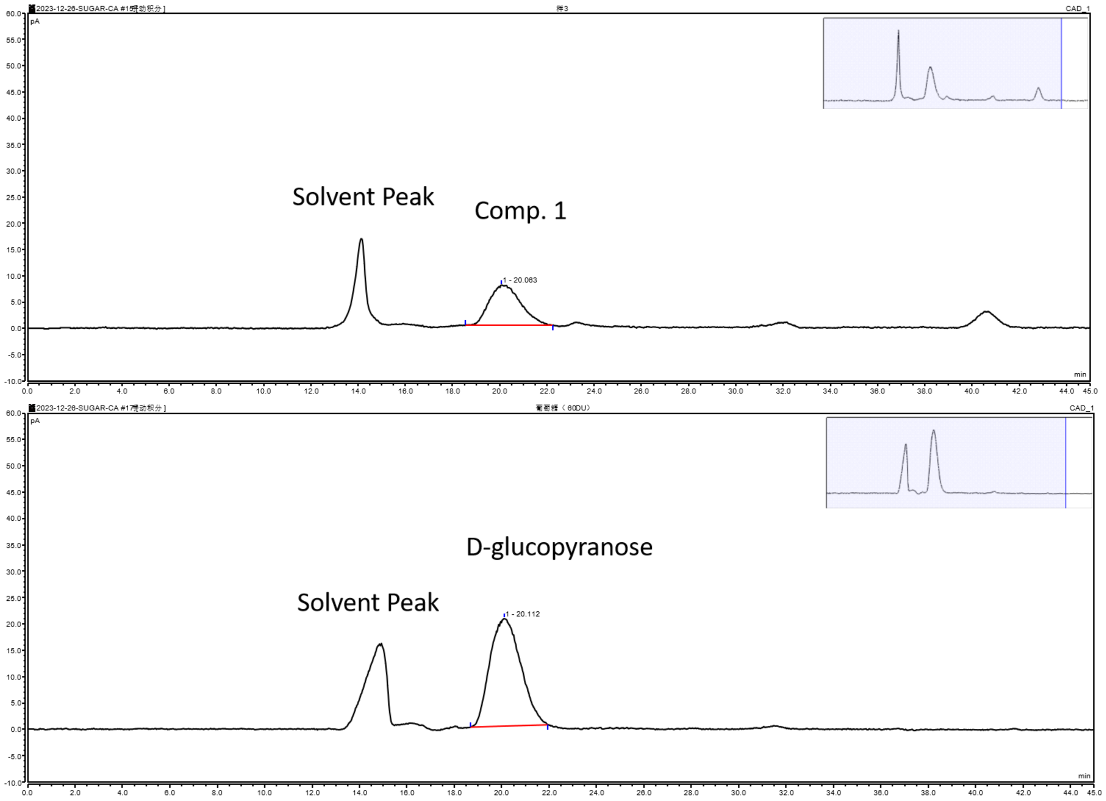

Figure S8. The HPLC pectrum of the compound **1** of D-glucopyranose

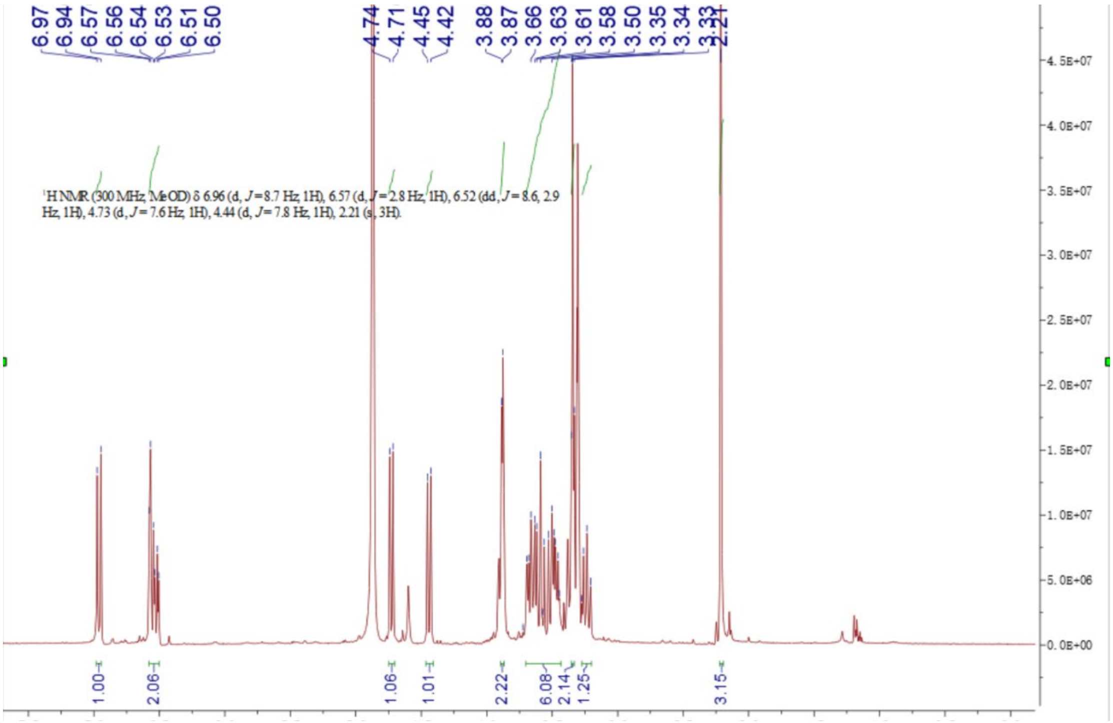

Figure S9. ^{1}H NMR spectrum of compound **2** (300 MHz, methanol-d_4)

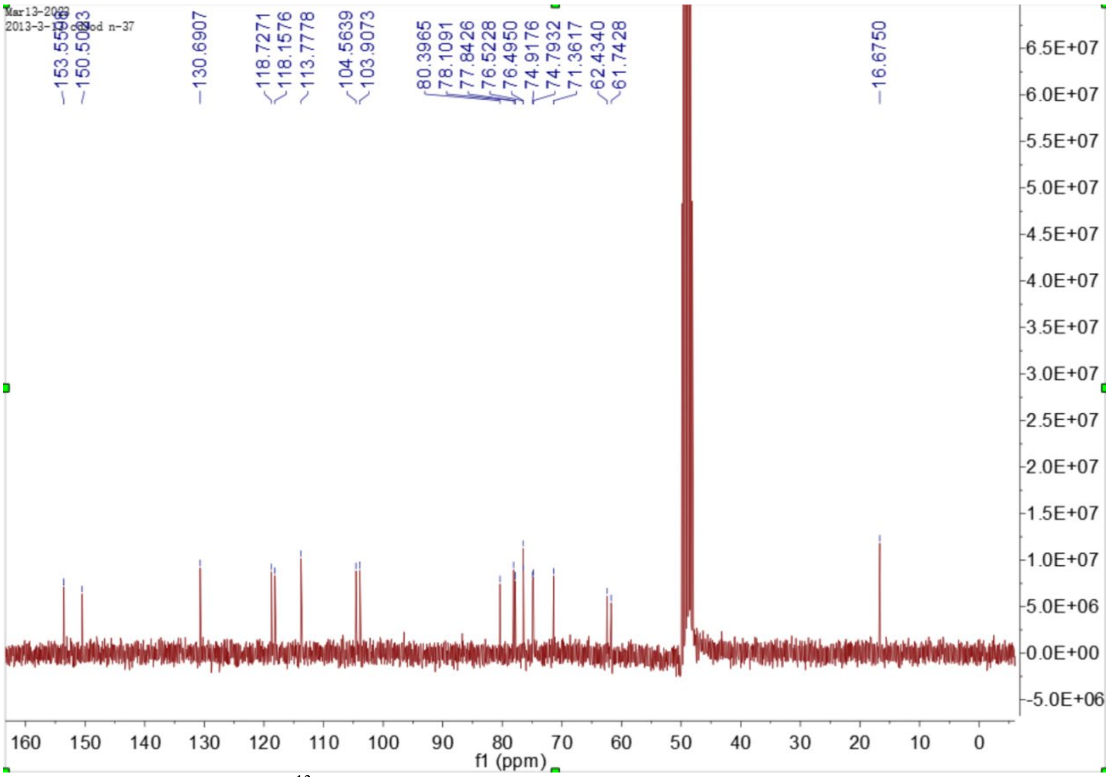

Figure S10. ^{13}C NMR spectrum of compound **2** (75 MHz, methanol-d_4)

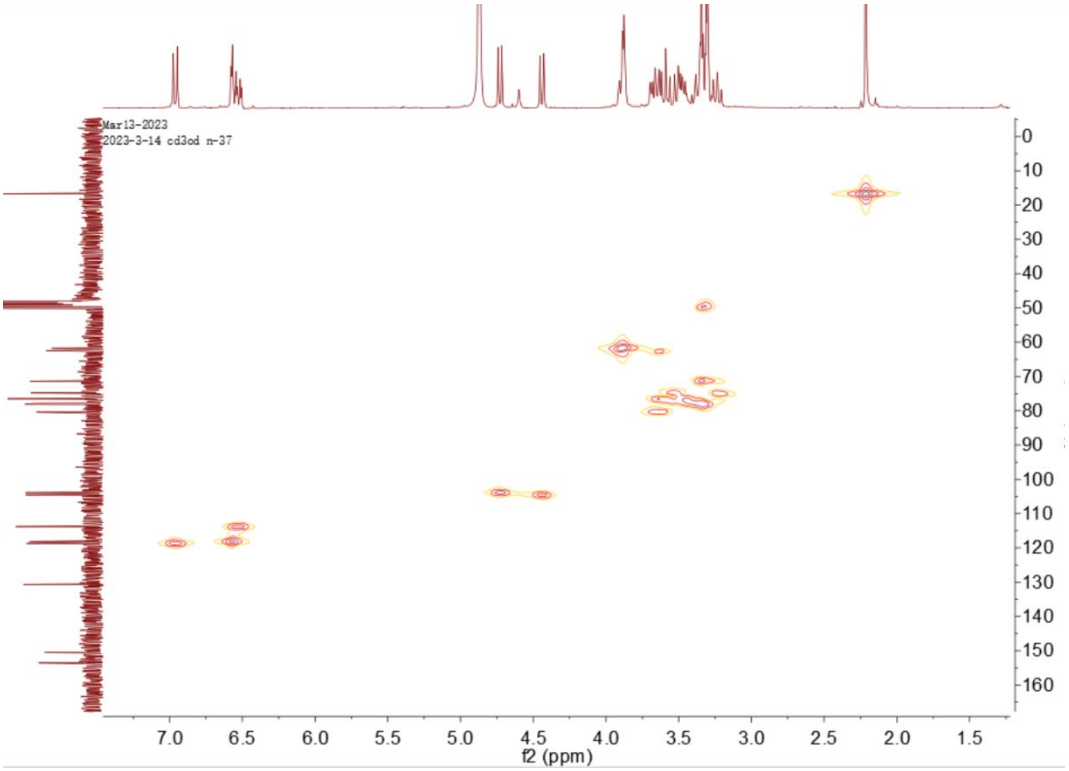

Figure S11. ^{1}H-^{13}C HMQC spectrum of compound **2** (methanol-d_4)

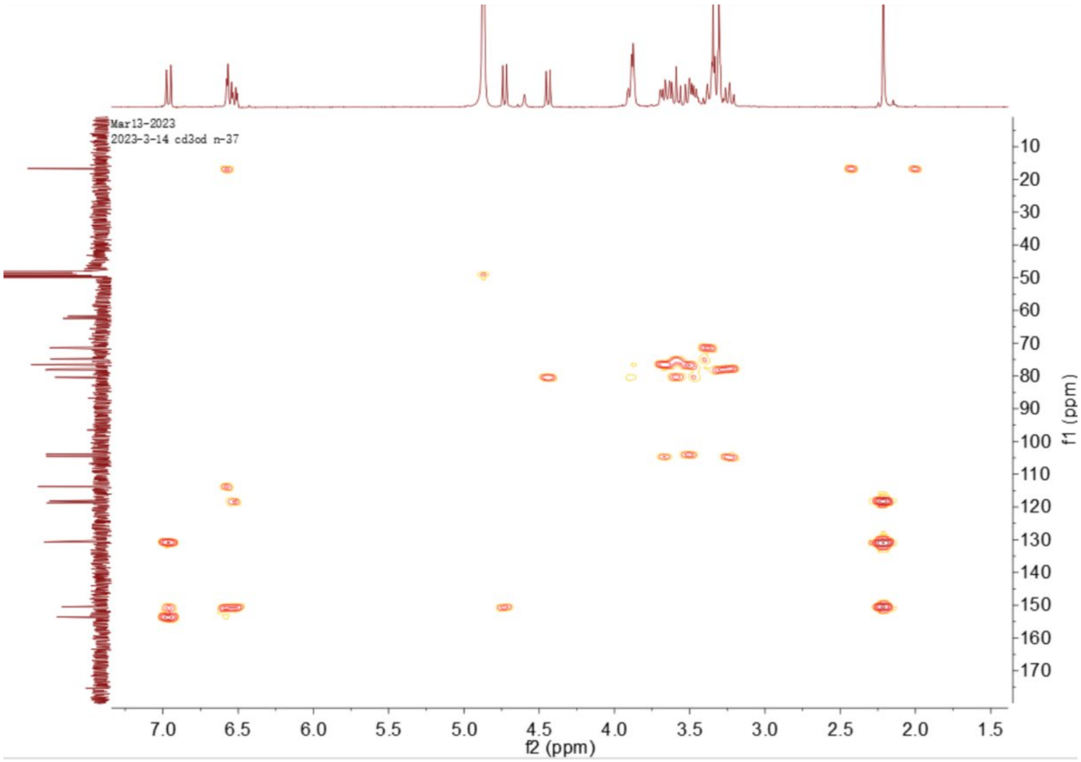

Figure S12. ^{1}H-^{13}C HMBC spectrum of compound **2** (methanol-d_4)

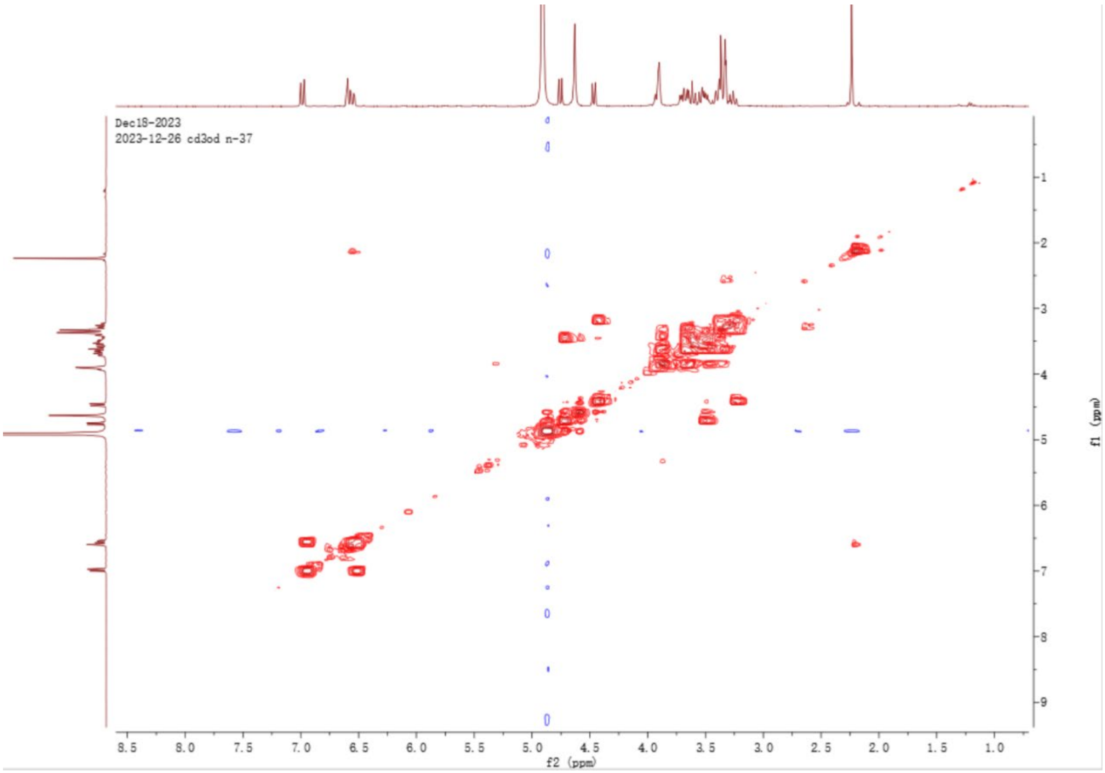

Figure S13. ^{1}H-^{1}H COSY spectrum of compound **2** (methanol-d_4)

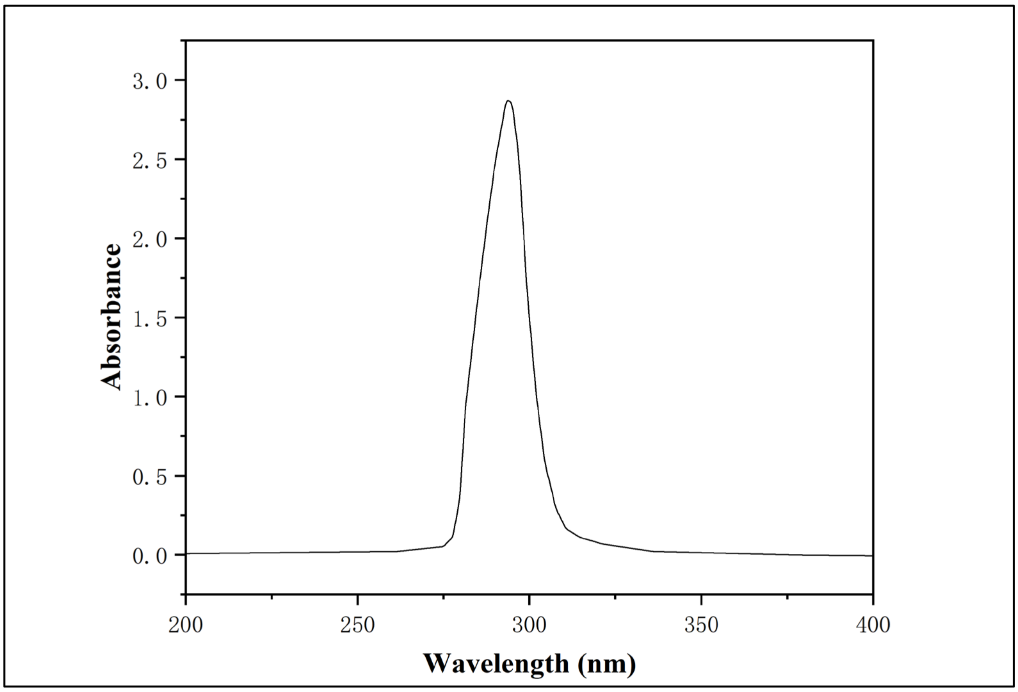

Figure S14. UV spectrum of compound **2**

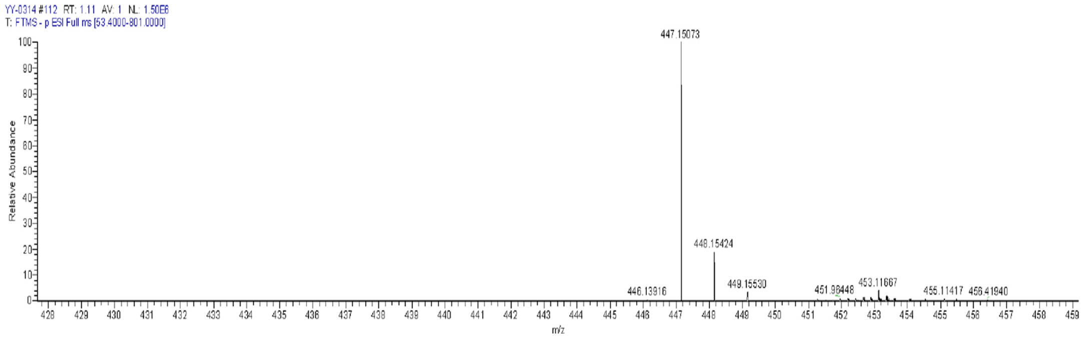

Figure S15. HRESIMS spectrum of compound **2**

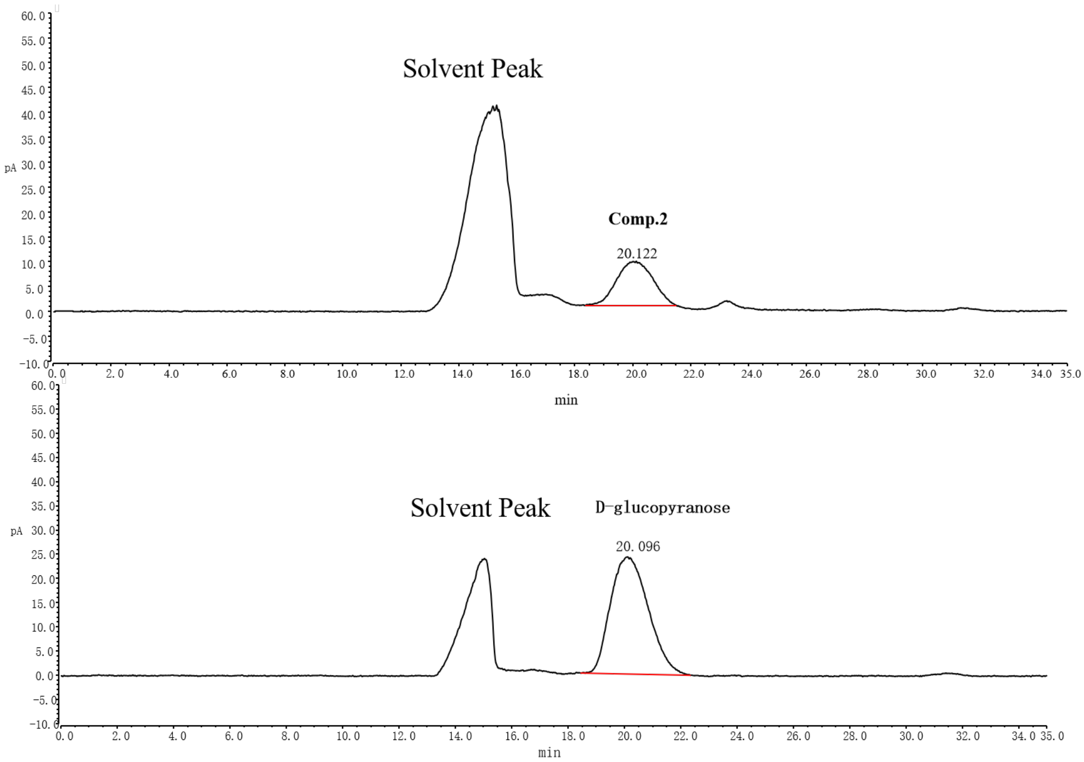

Figure S16. The HPLC pectrum of the compound **2** of D-glucopyranose

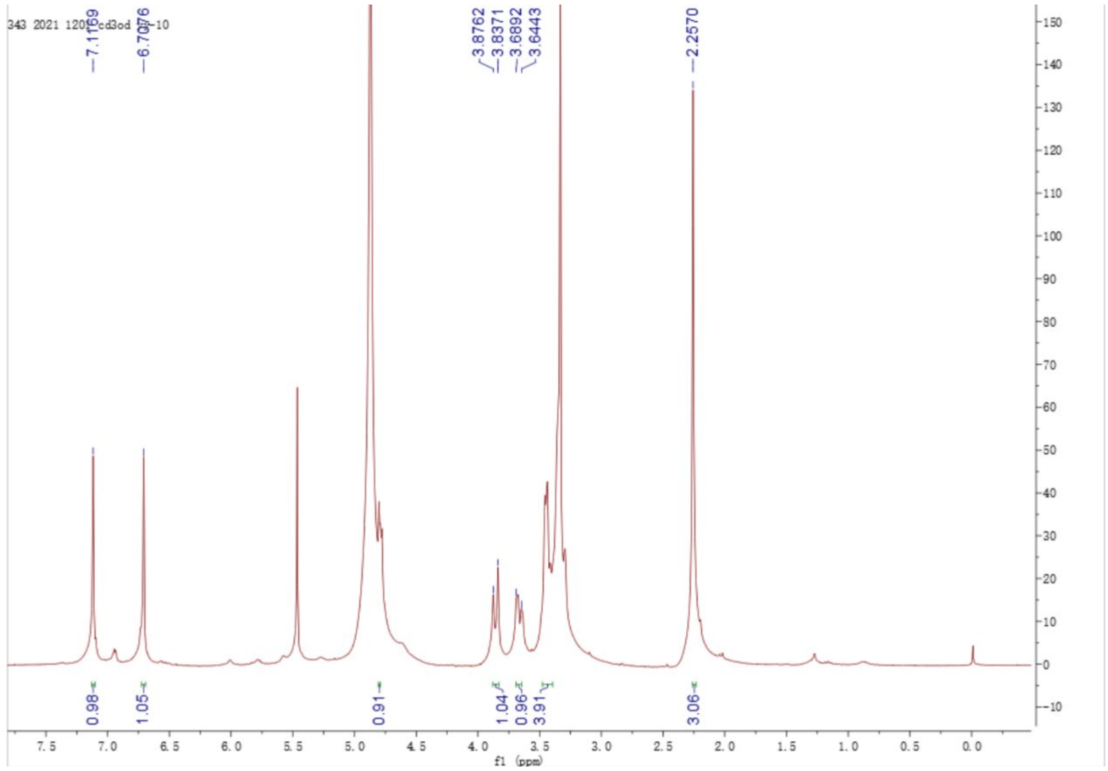

Figure S17. ^{1}H NMR spectrum of compound **3** (300 MHz, methanol-d_4)

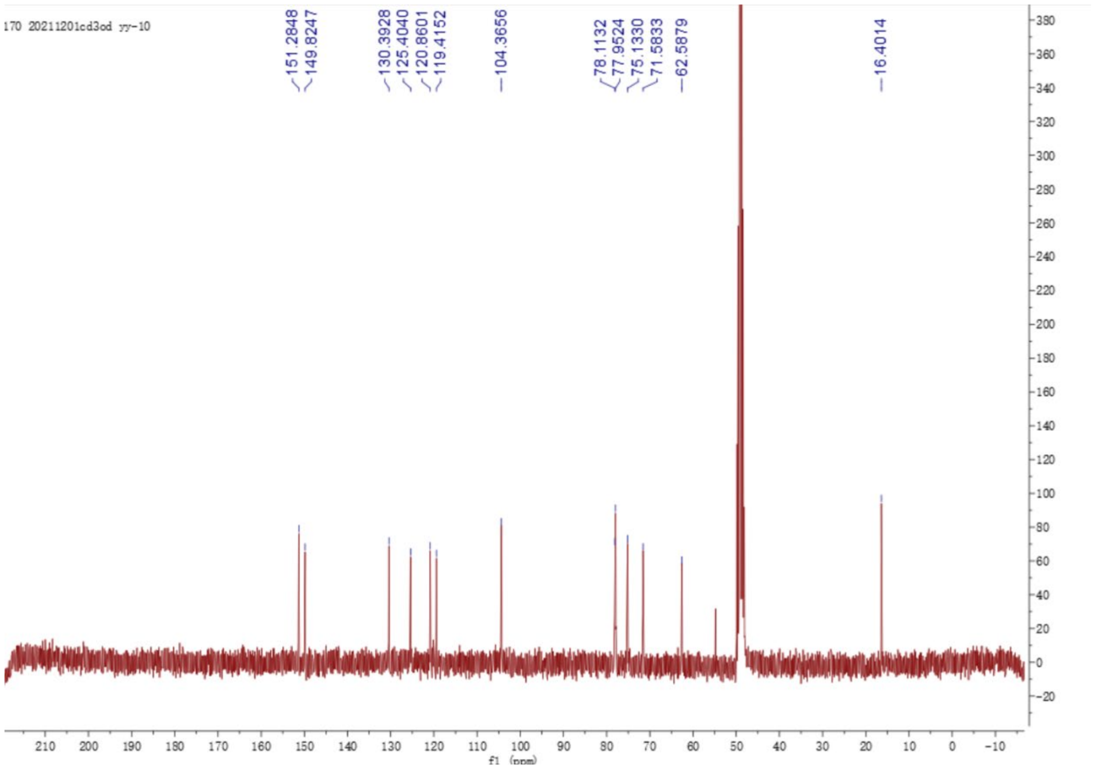

Figure S18. ^{13}C NMR spectrum of compound **3** (75 MHz, methanol-d_4)

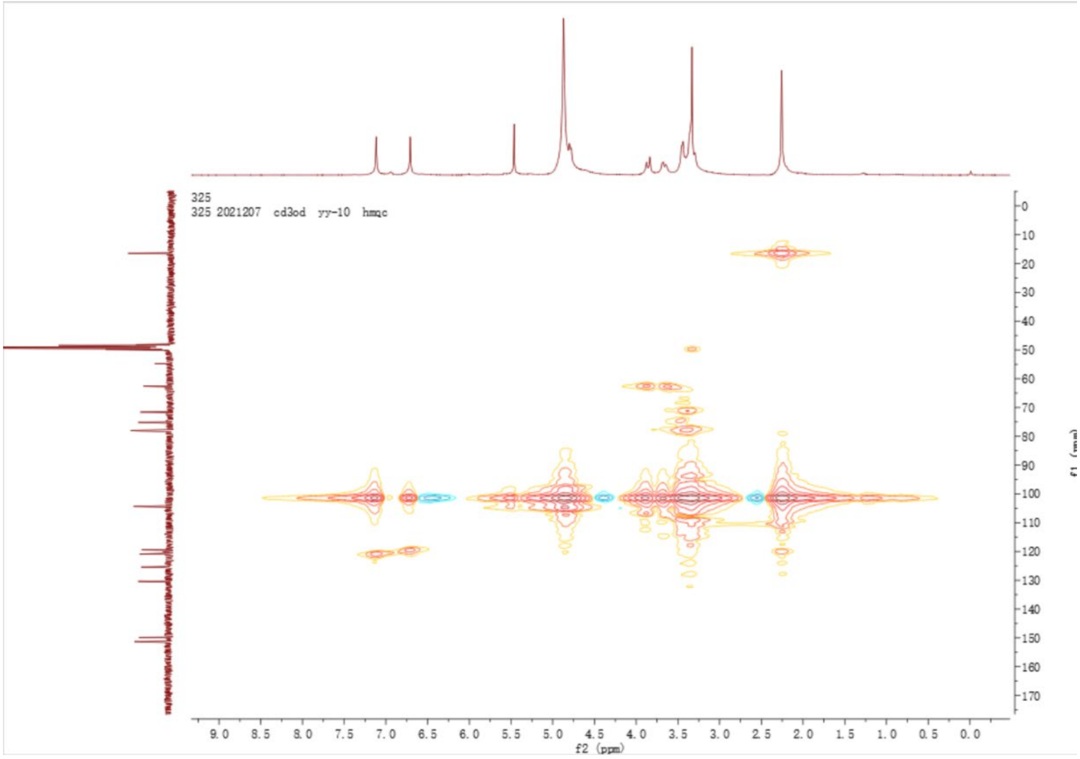

Figure S19. ^{1}H-^{13}C HMQC spectrum of compound **3** (methanol-d_4)

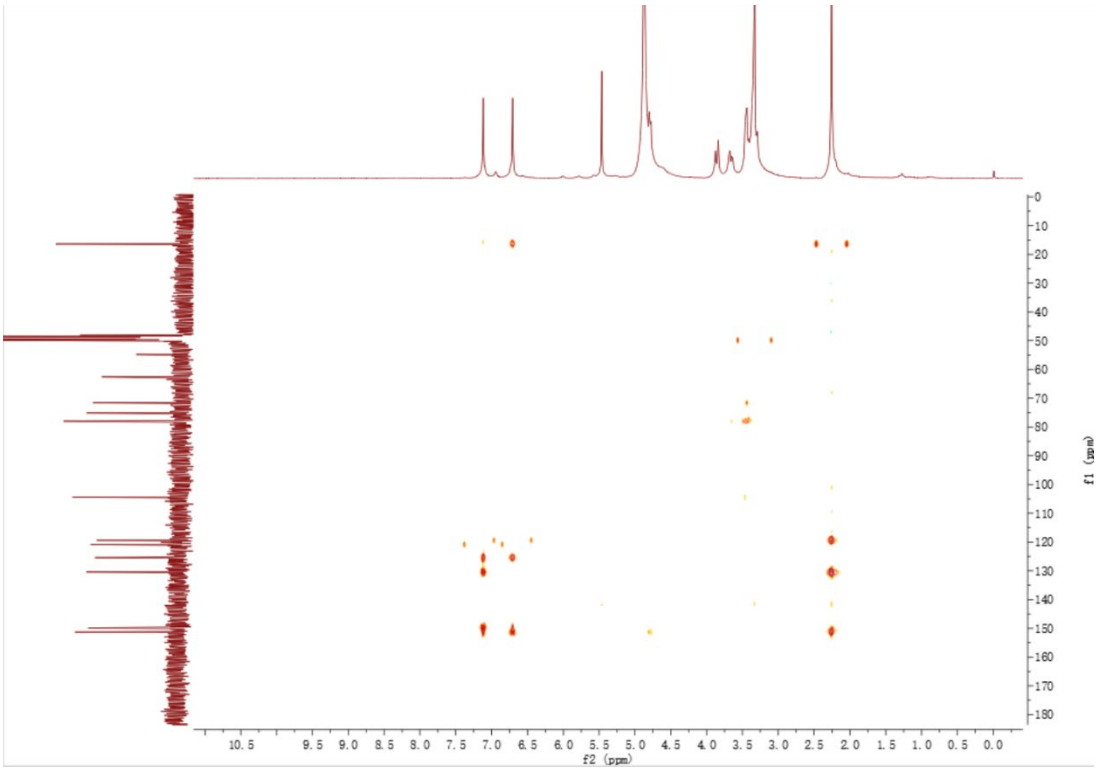

Figure S20. ^{1}H-^{13}C HMBC spectrum of compound **3** (methanol-d_4)

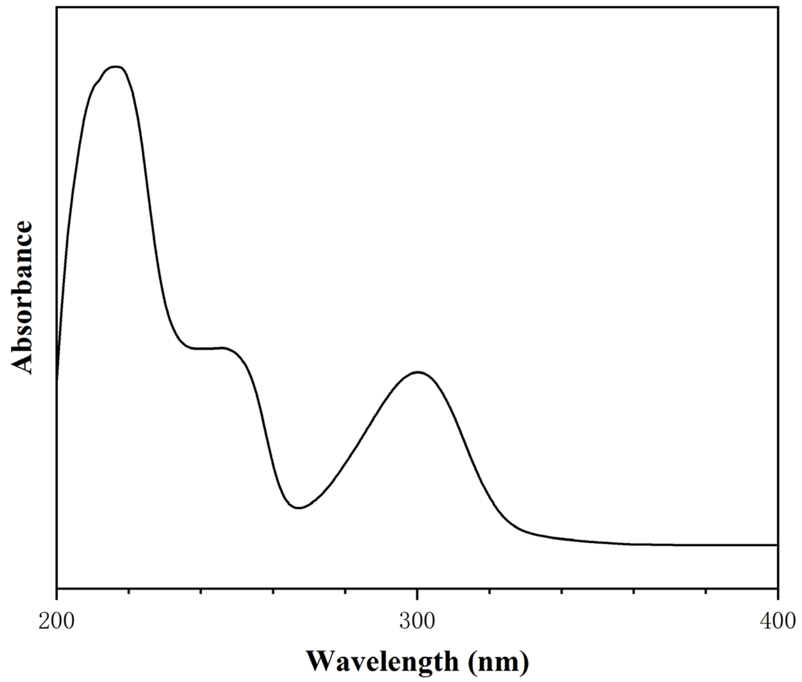

Figure S21. UV spectrum of compound **3**

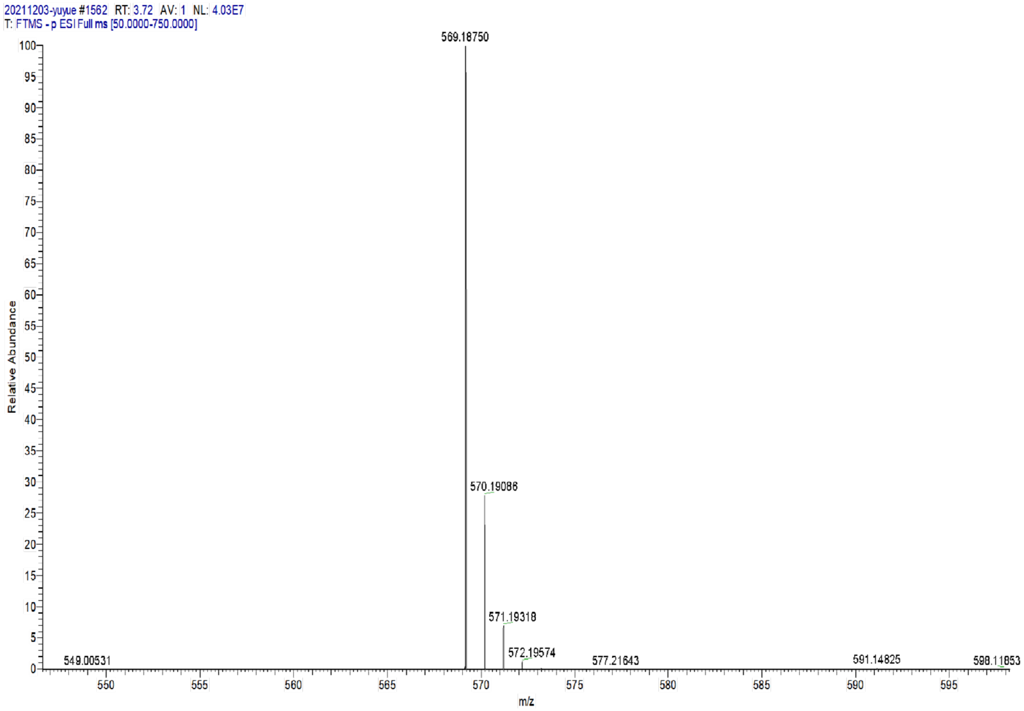

Figure S22. HRESIMS spectrum of compound **3**

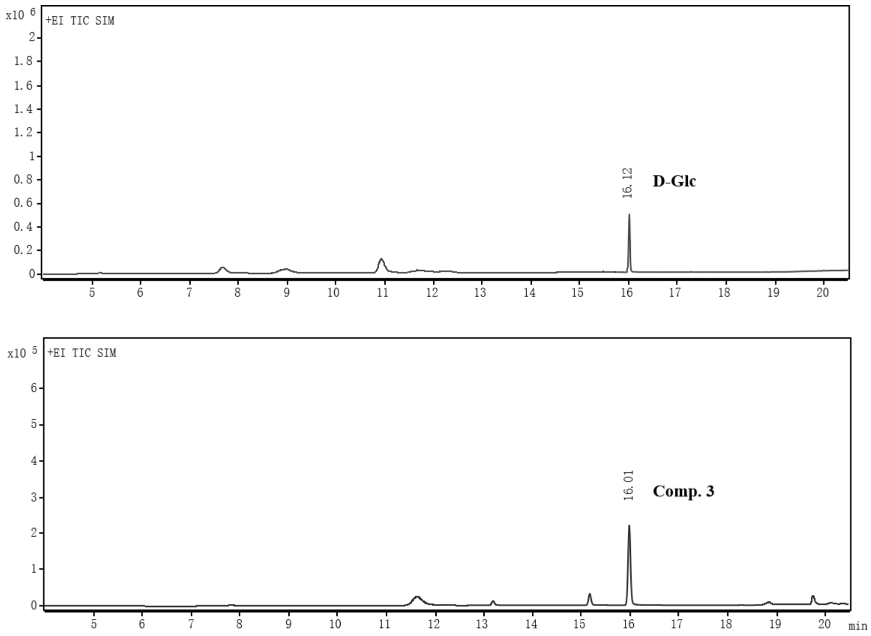

Figure S23. The GC pectrum of the compound **3** of D-glucopyranose

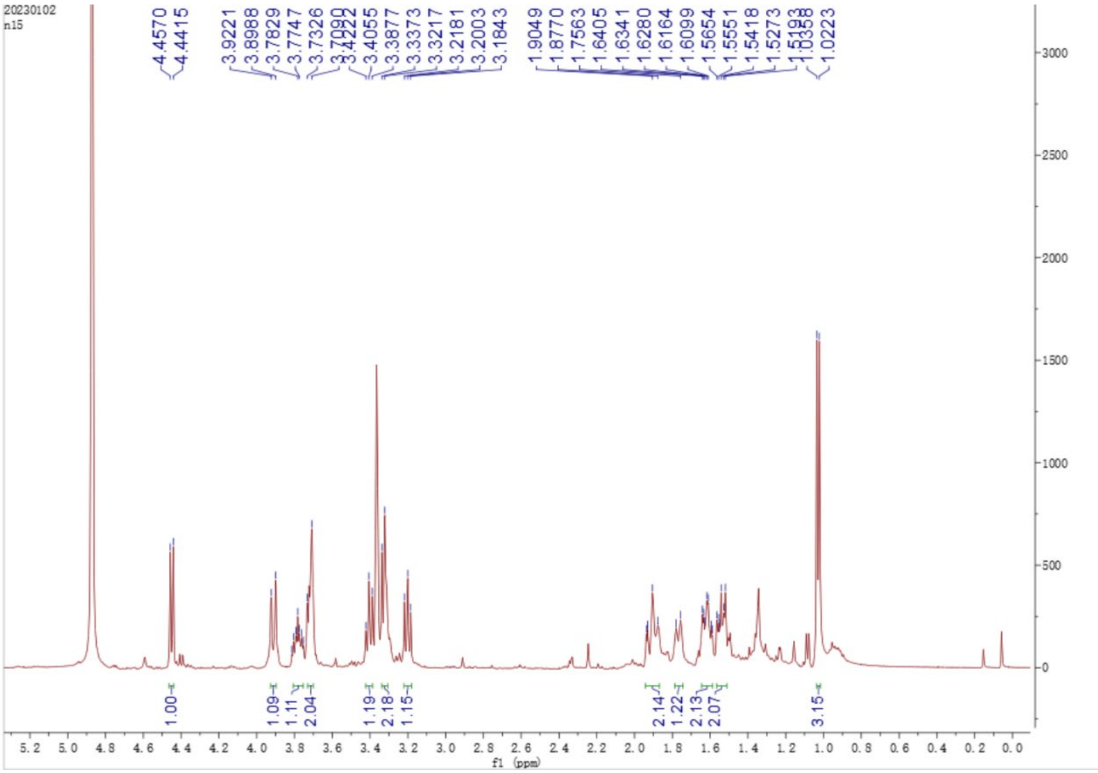

Figure S24. ^{1}H NMR spectrum of compound **4** (300 MHz, methanol-d_4)

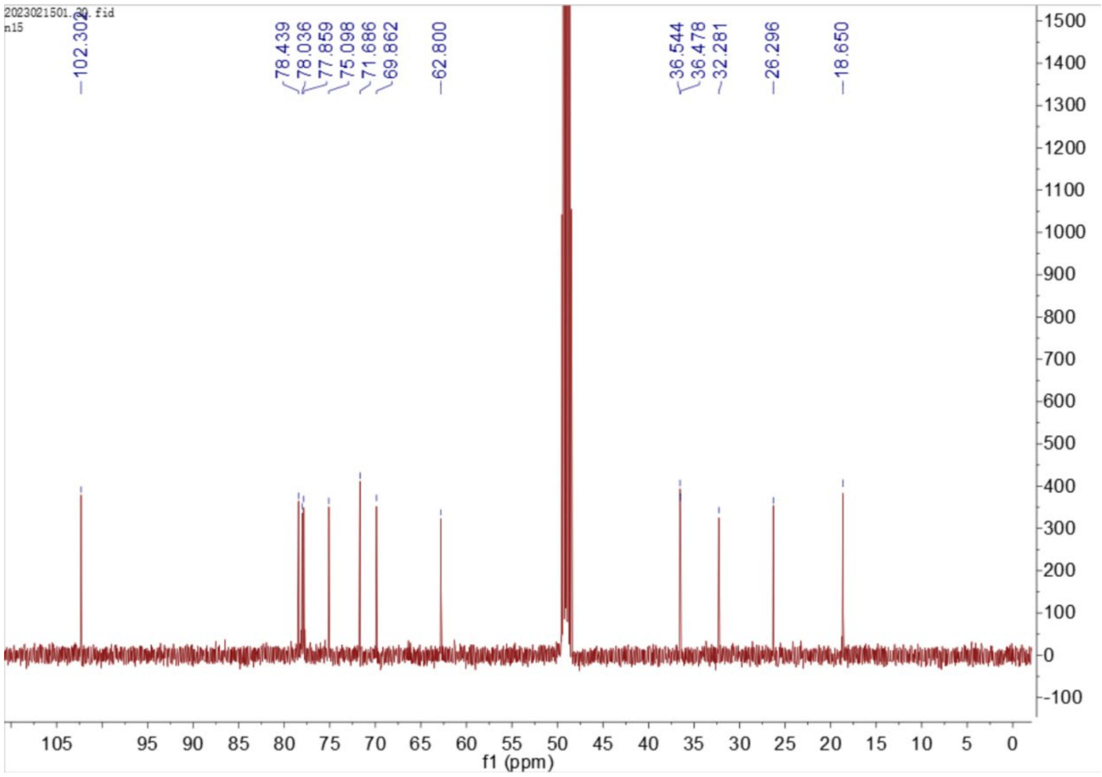

Figure S25. ^{13}C NMR spectrum of compound **4** (75 MHz, methanol-d_4)

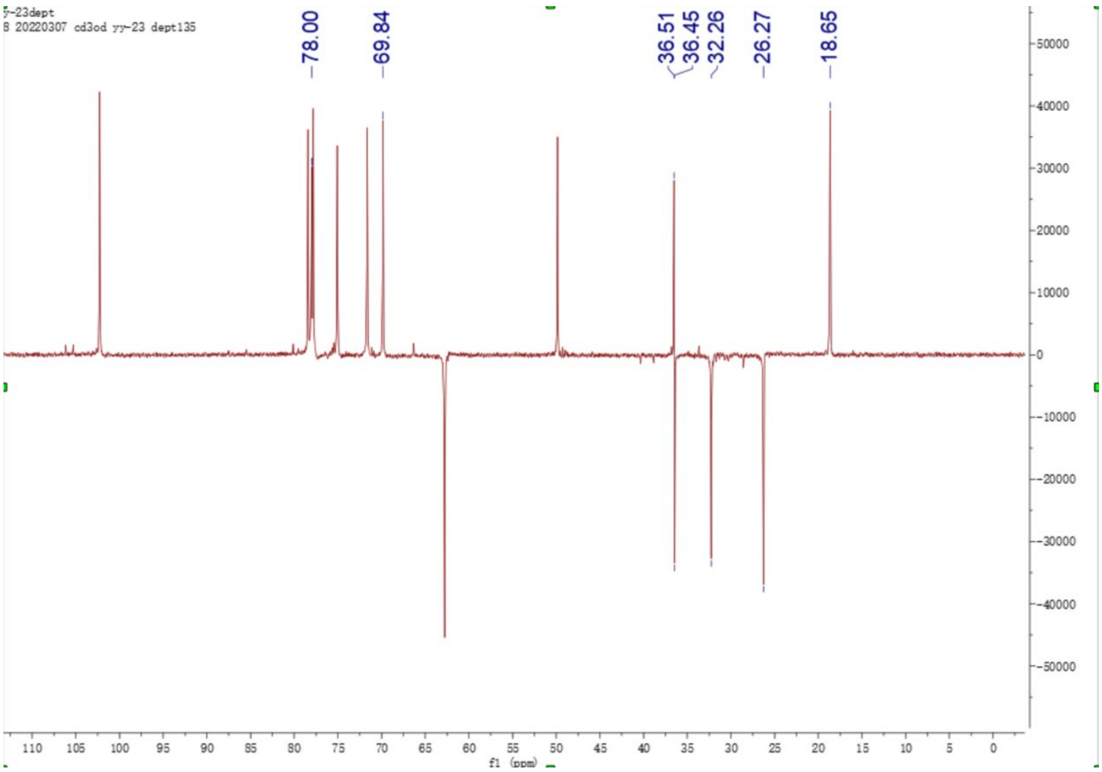

Figure S26. DEPT spectrum of compound **4** in methanol-d_4

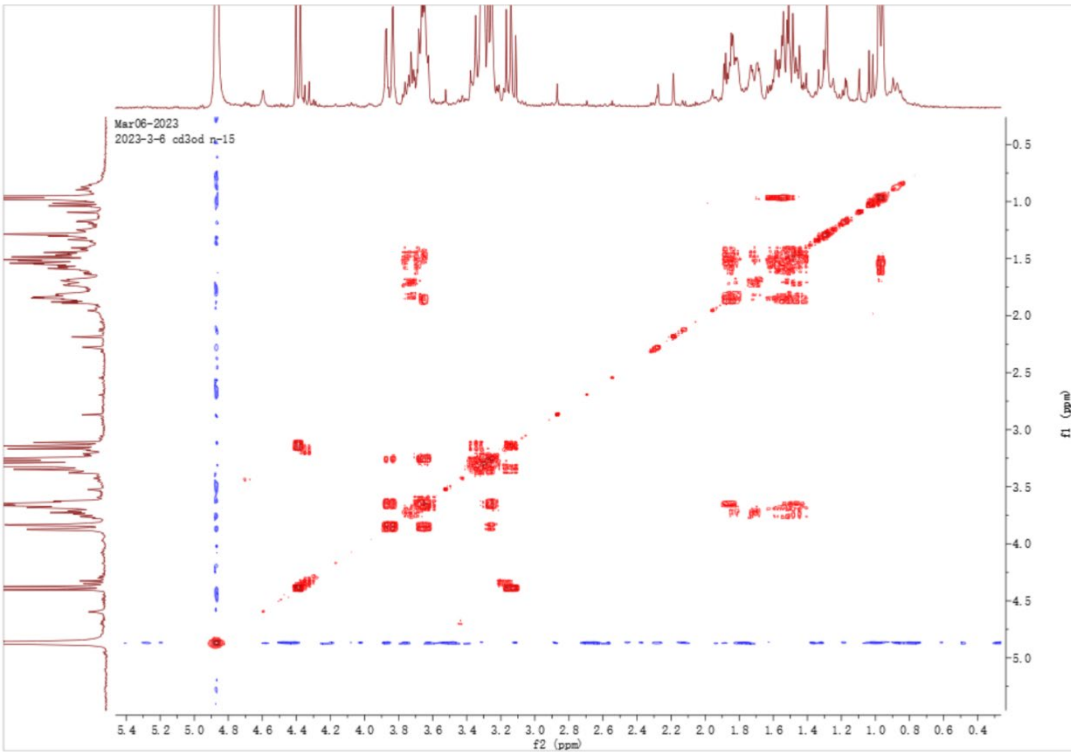

Figure S27. ^{1}H-^{1}H COSY spectrum of compound **4** (methanol-d_4)

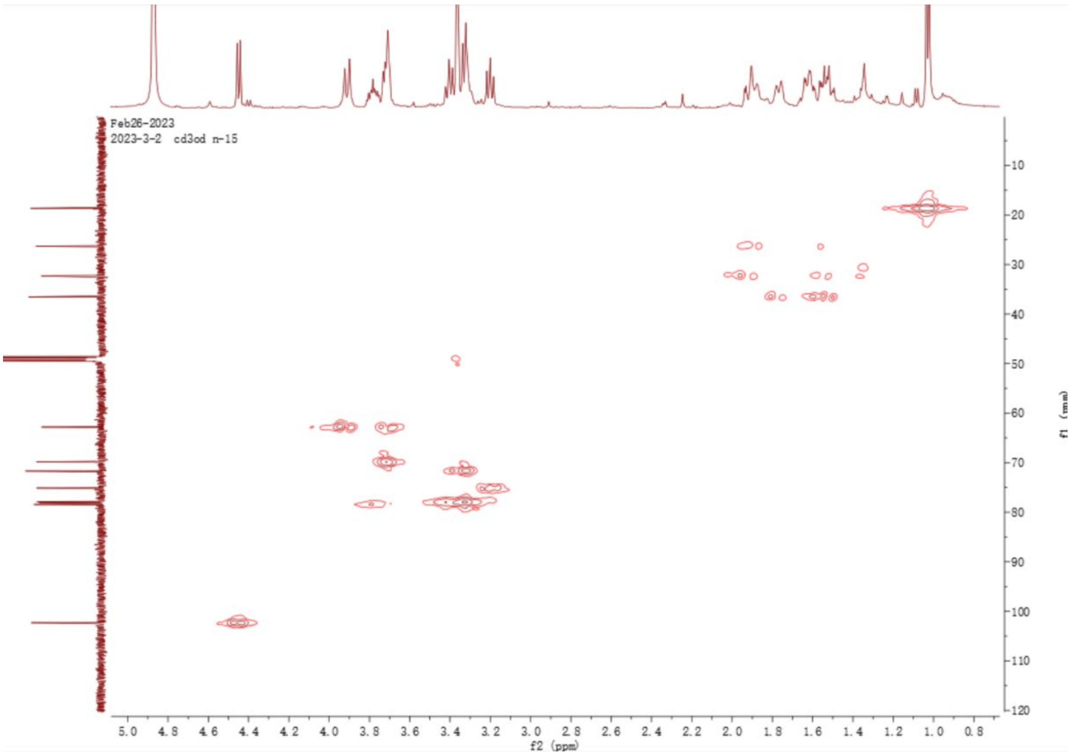

Figure S28. ^{1}H-^{13}C HMQC spectrum of compound **4** (methanol-d_4)

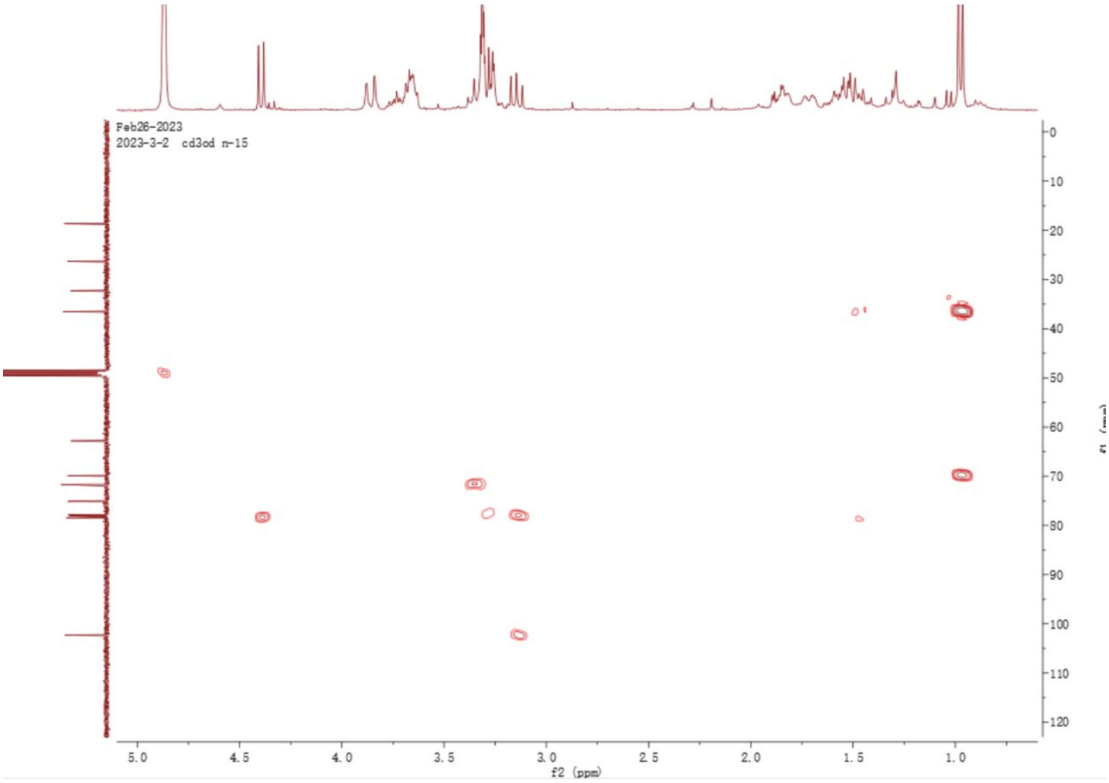

Figure S29. ^{1}H-^{13}C HMBC spectrum of compound **4** (methanol-d_4)

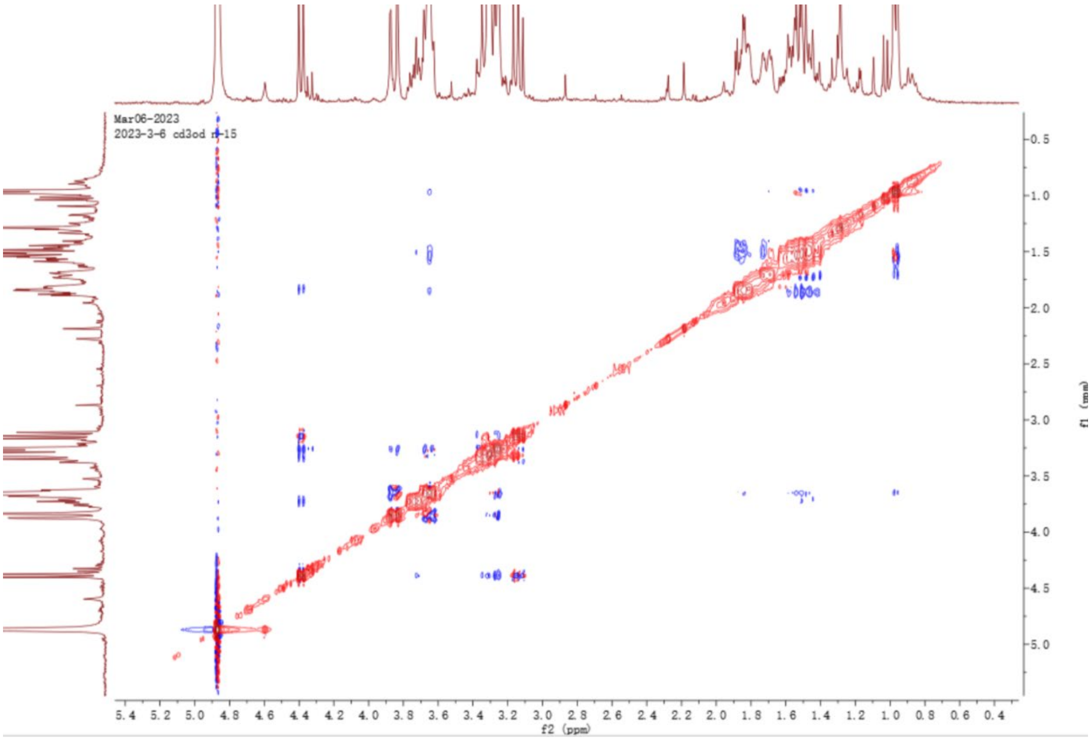

Figure S30. NOESY spectrum of compound **4** in methanol-d_4

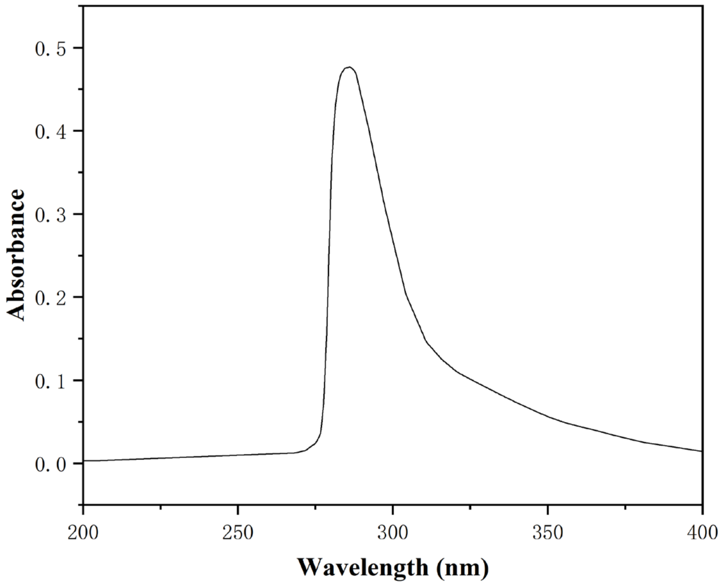

Figure S31. UV spectrum of compound **4**

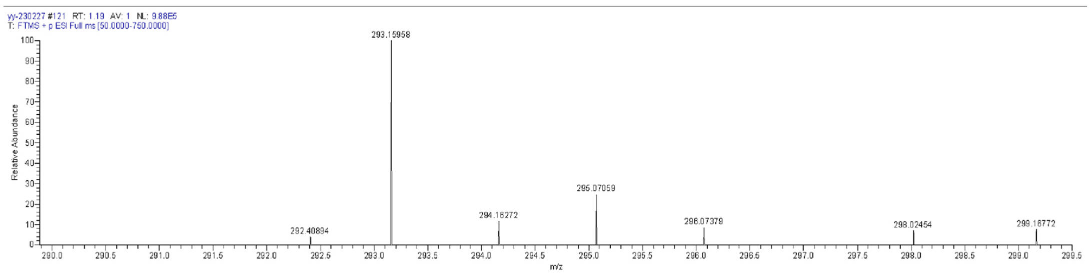

Figure S32. HRESIMS spectrum of compound **4**

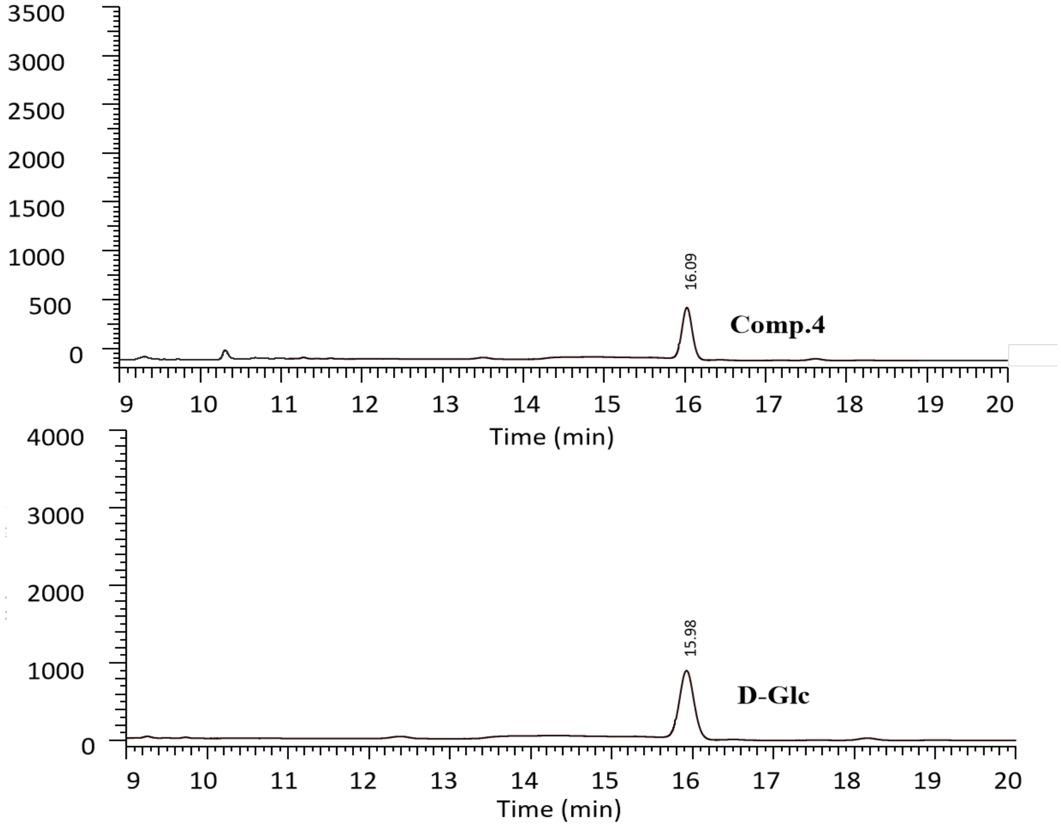

Figure S33. The HPLC pectrum of the compound **4** of D-glucopyranose

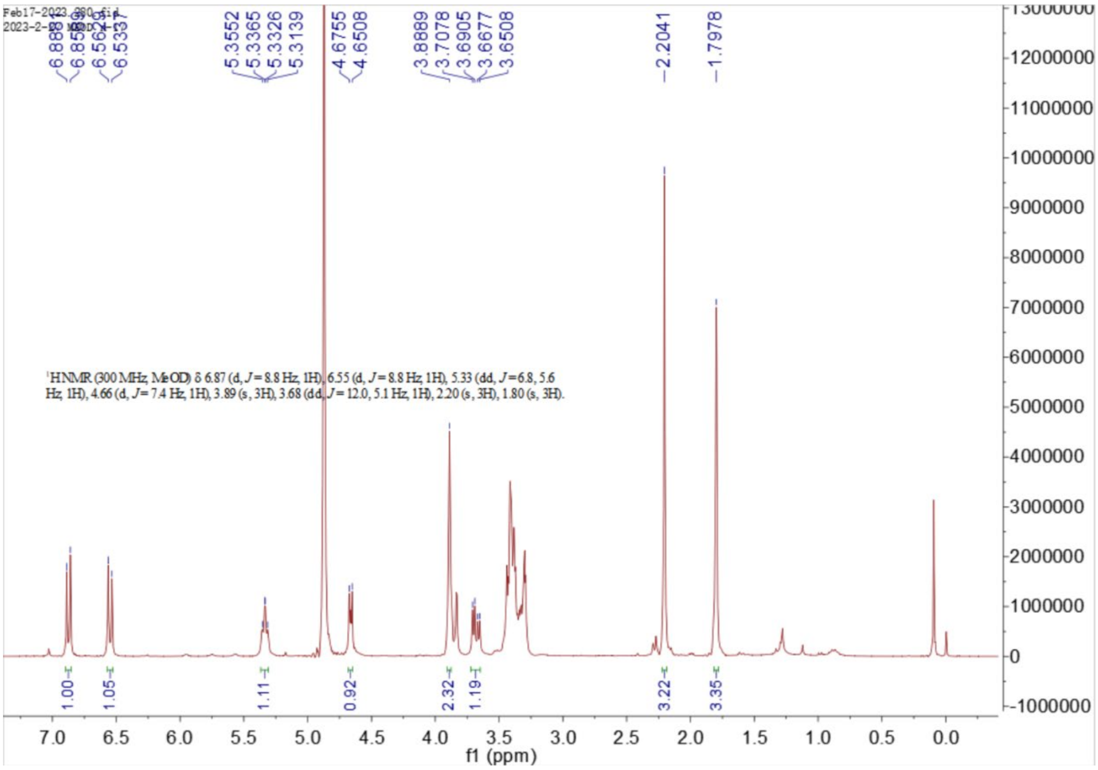

Figure S34. ^{1}H NMR spectrum of compound **5** (300 MHz, methanol-d_4)

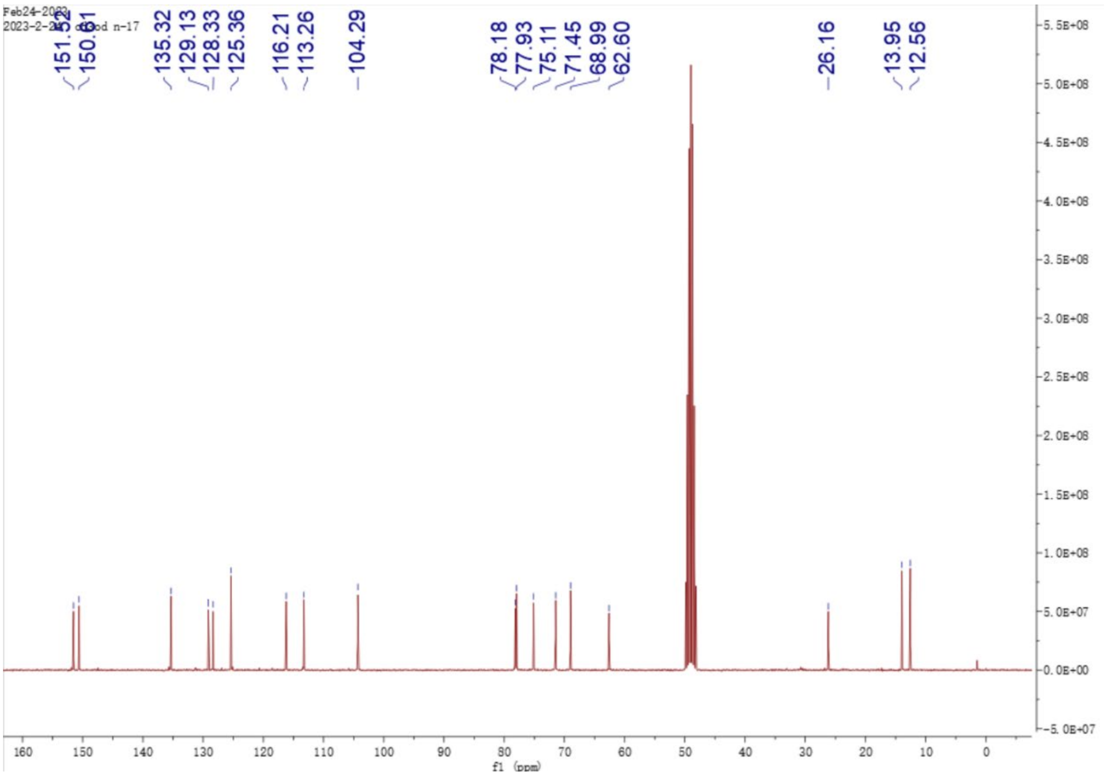

Figure S35. ^{13}C NMR spectrum of compound **5** (75 MHz, methanol-d_4)

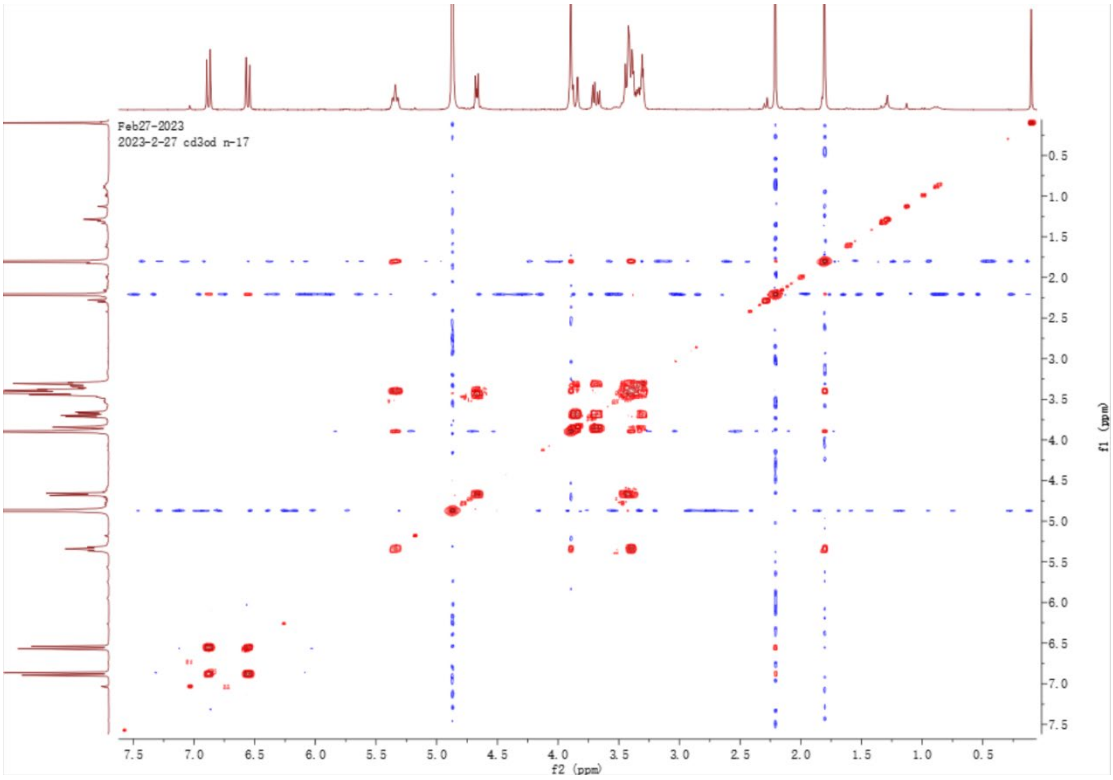

Figure S36. ^{1}H-^{1}H COSY spectrum of compound **5** (methanol-d_4)

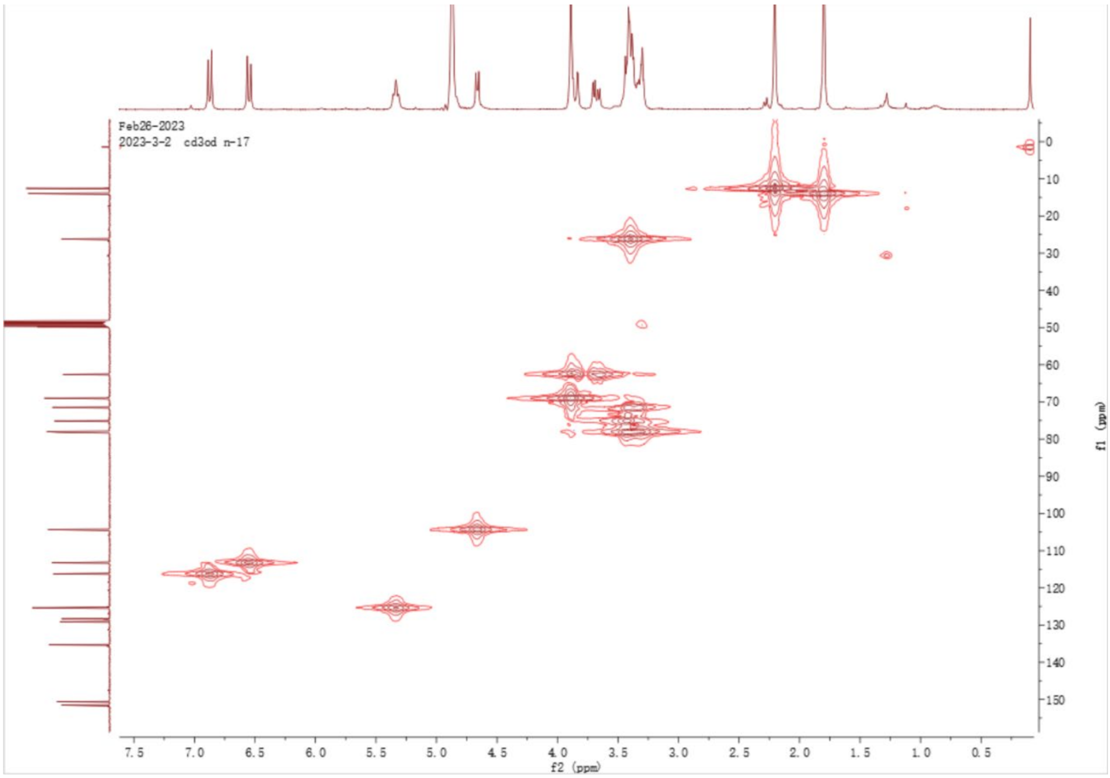

Figure S37. ^{1}H-^{13}C HMQC spectrum of compound **5** (methanol-d_4)

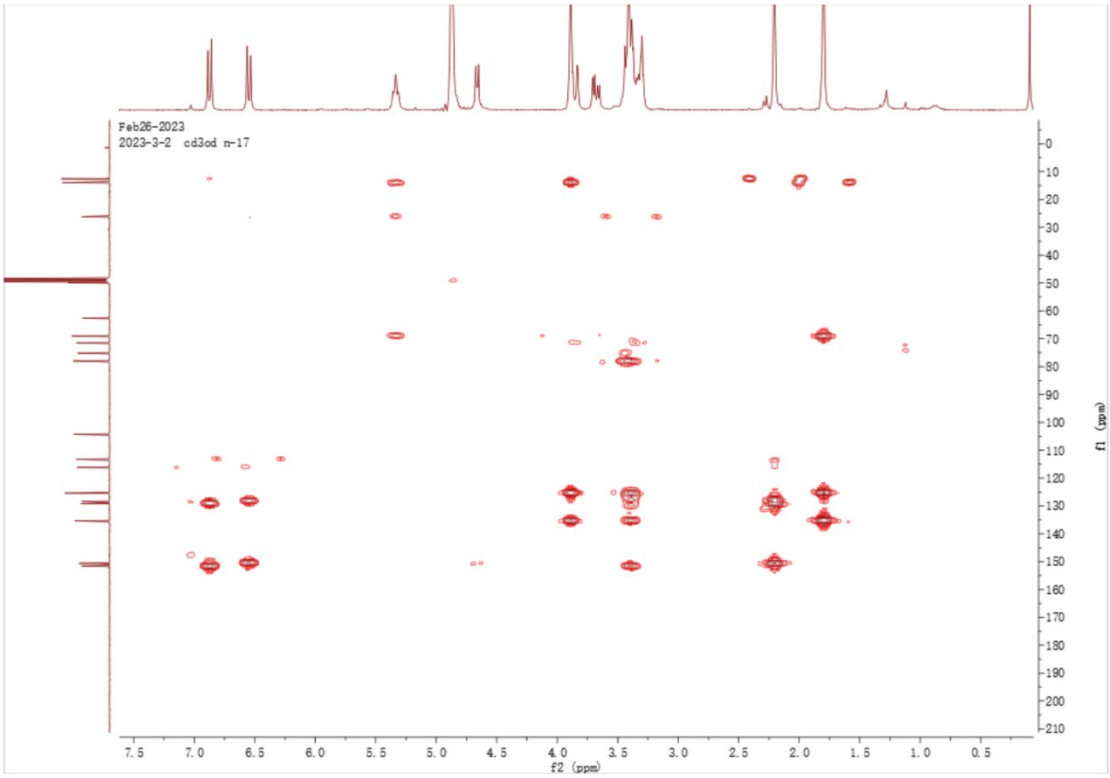

Figure S38. ^{1}H-^{13}C HMBC spectrum of compound **5** (methanol-d_4)

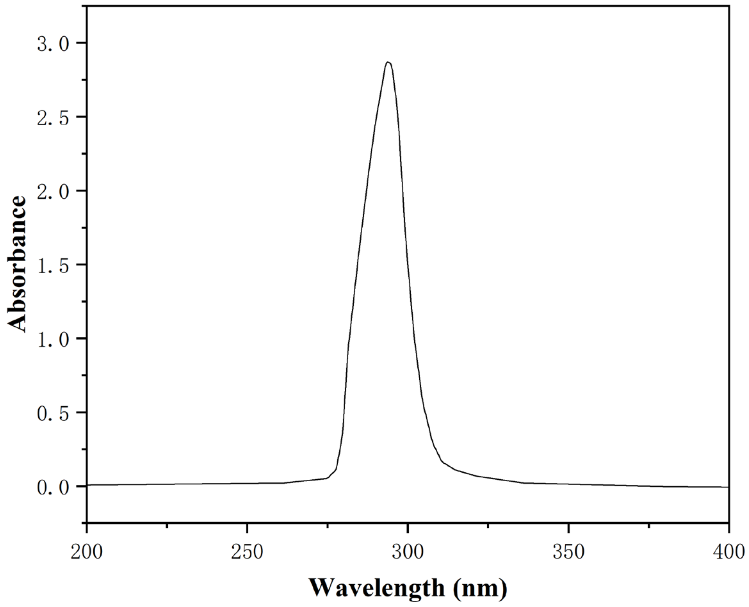

Figure S39. UV spectrum of compound **5**

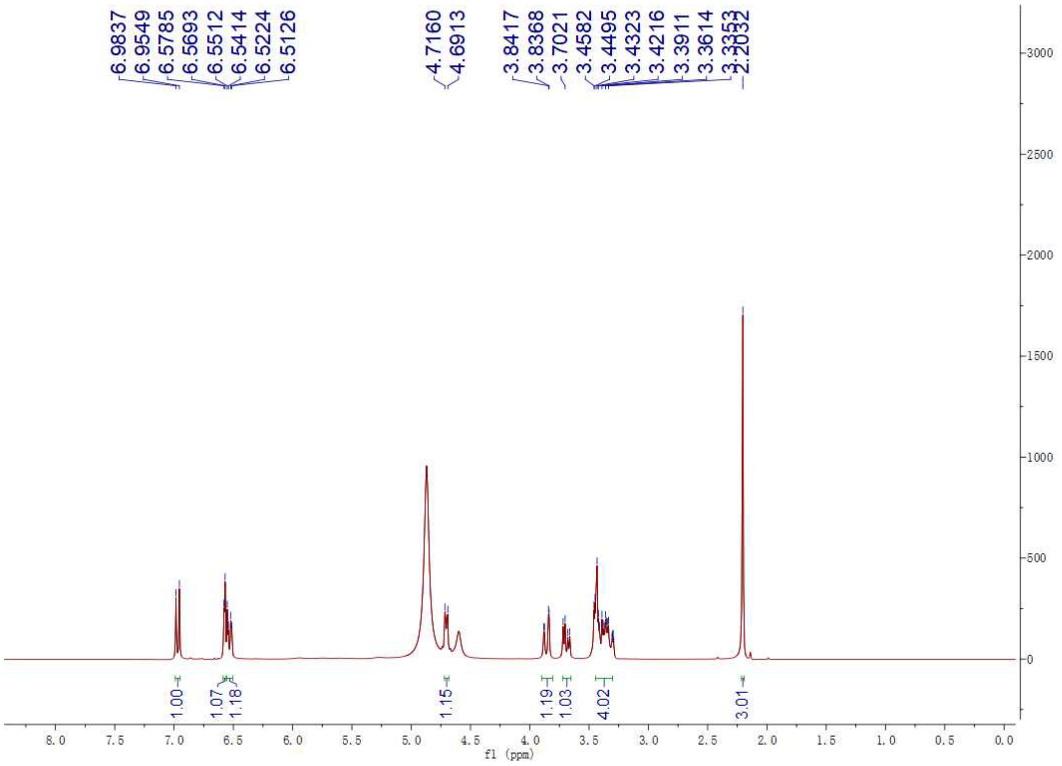

Figure S40. ^{1}H-NMR spectrum of compound **6** (300 MHz, methanol-d_4)

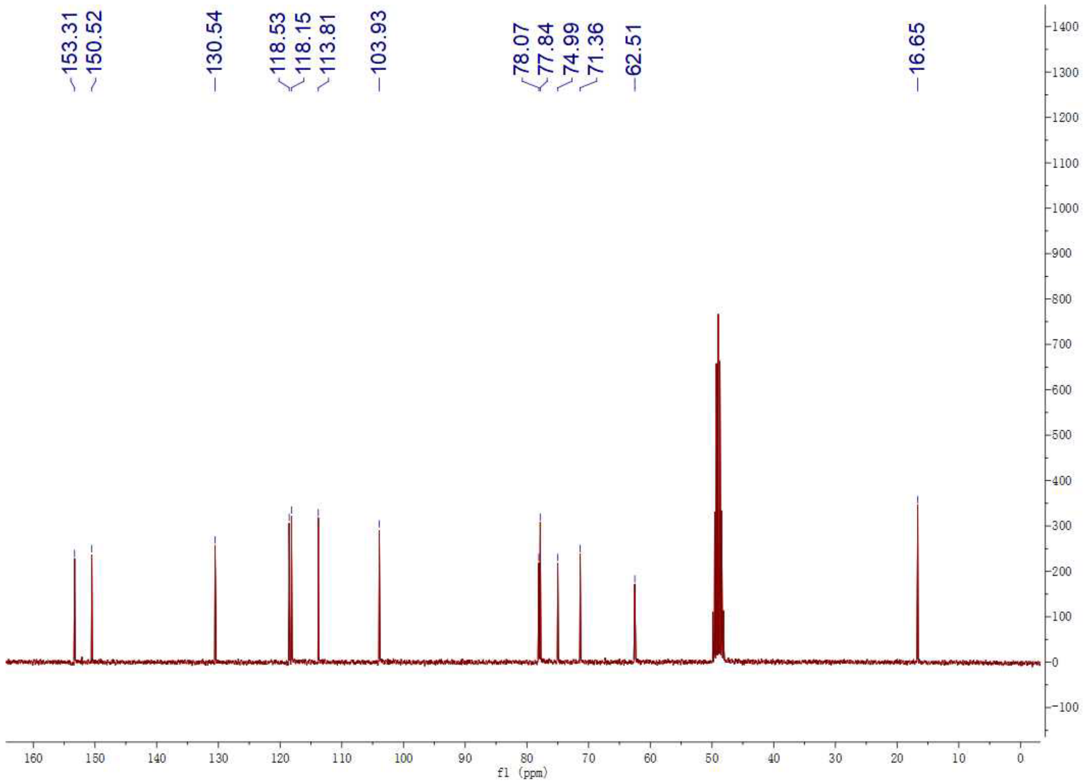

Figure S41. ^{13}C-NMR spectrum of compound **6** (75 MHz, methanol-d_4)

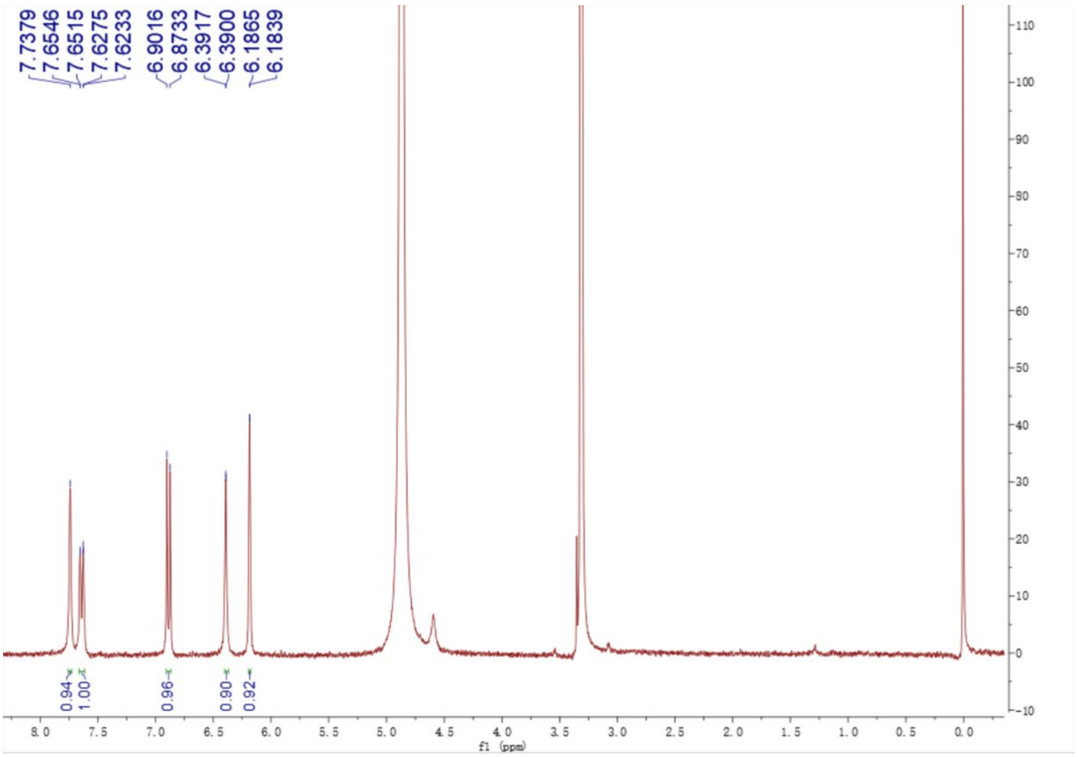

Figure S42. ^{1}H-NMR spectrum of compound **7** (300 MHz, methanol-d_4)

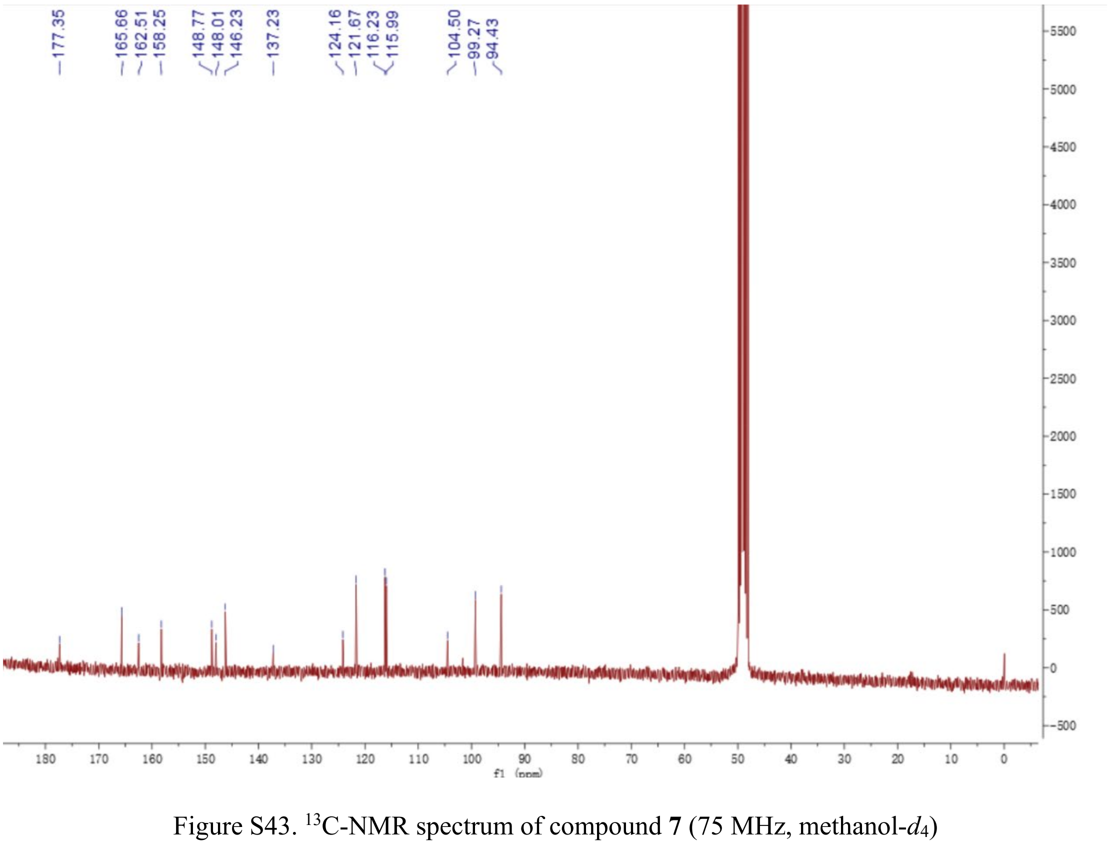

Figure S43. ^{13}C-NMR spectrum of compound **7** (75 MHz, methanol-d_4)

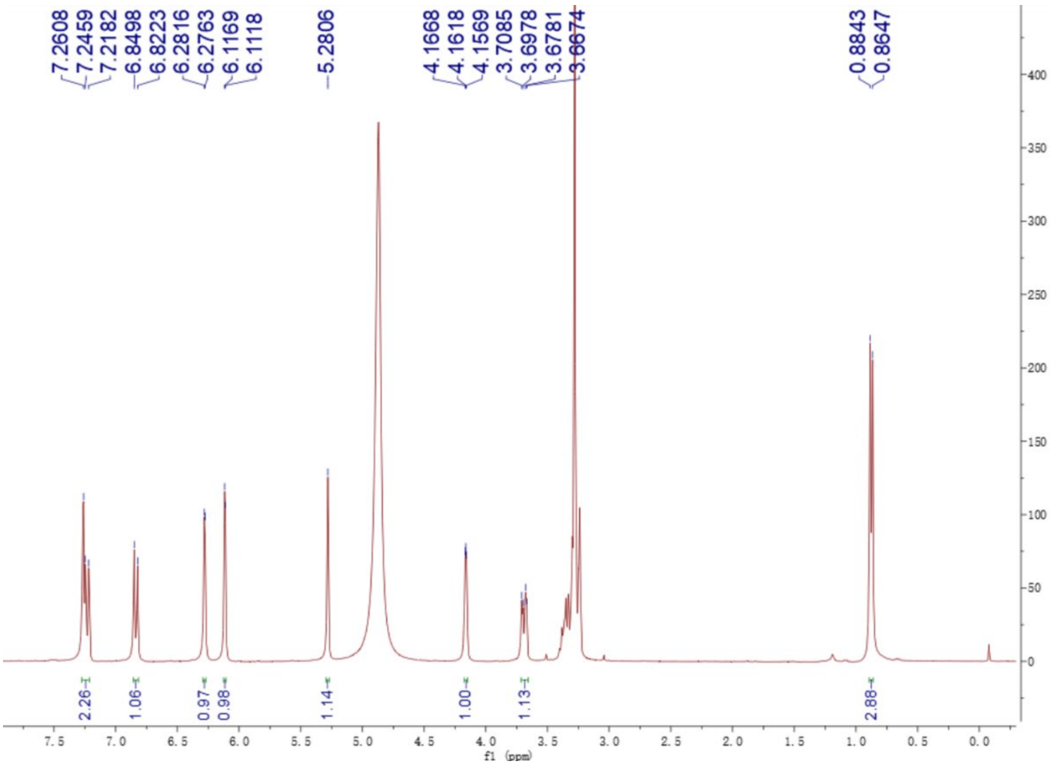

Figure S44. ^{1}H-NMR spectrum of compound **8** (300 MHz, methanol-d_4)

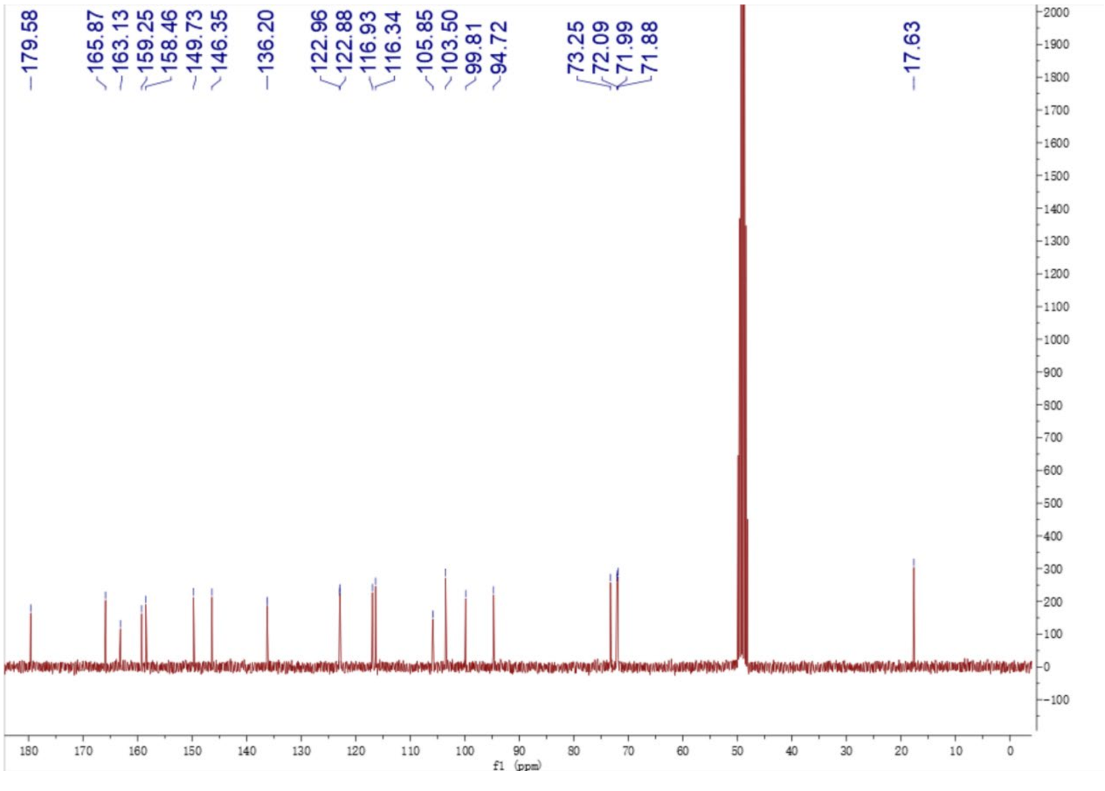

Figure S45. ^{13}C-NMR spectrum of compound **8** (75 MHz, methanol-d_4)

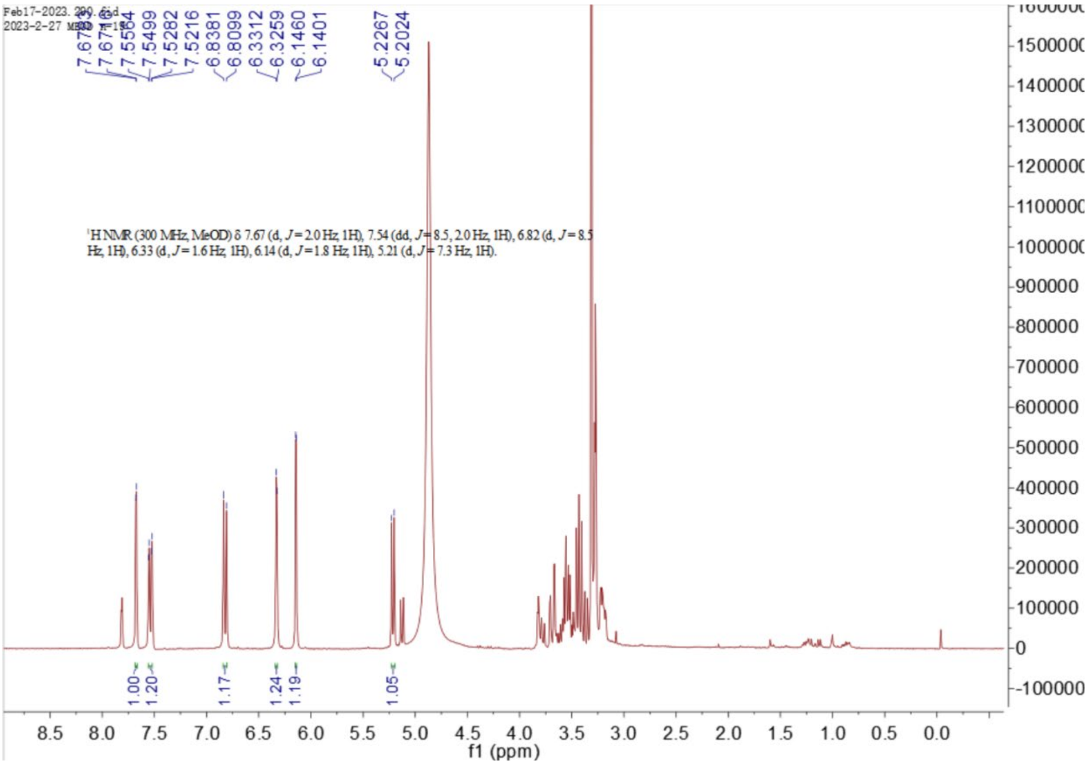

Figure S46. ^{1}H-NMR spectrum of compound **9** (300 MHz, methanol-d_4)

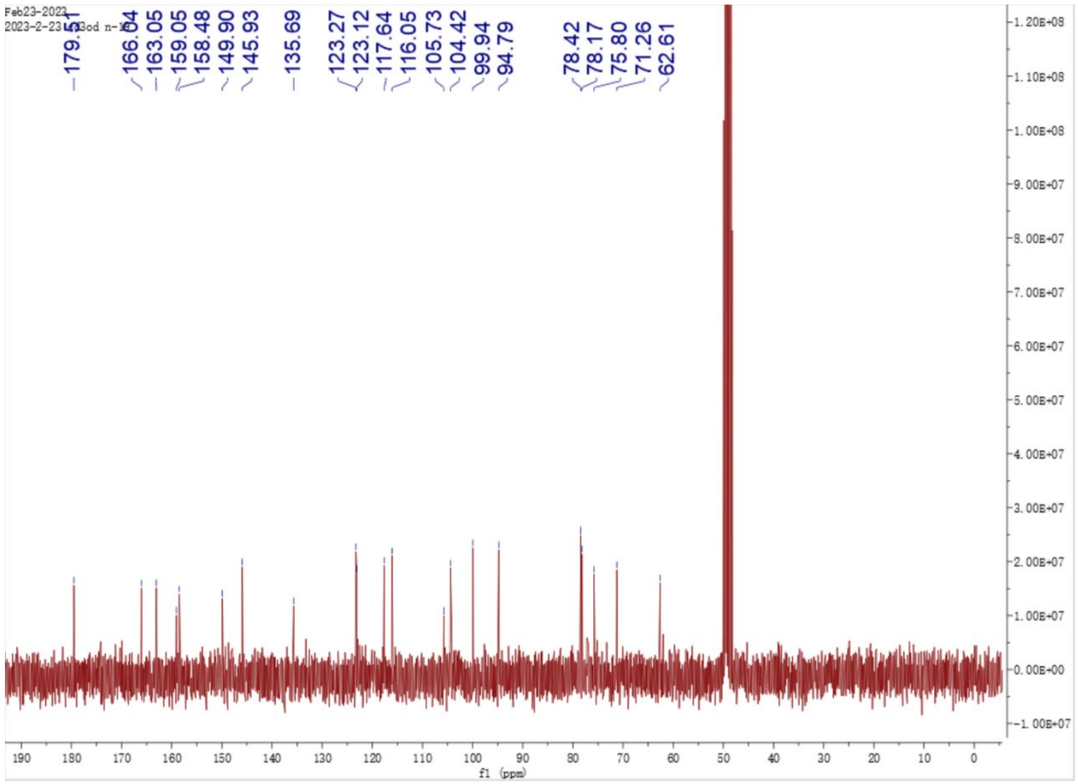

Figure S47. ^{13}C-NMR spectrum of compound **9** (75 MHz, methanol-d_4)

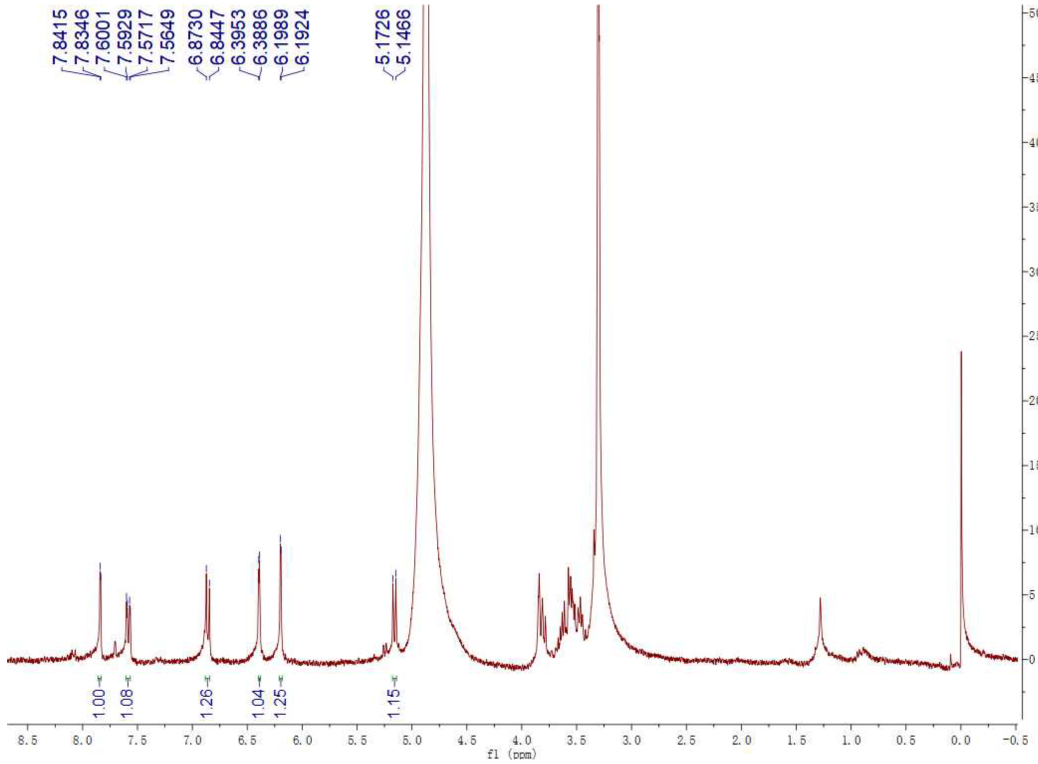

Figure S48. ^{1}H-NMR spectrum of compound **10** (300 MHz, methanol-d_4)

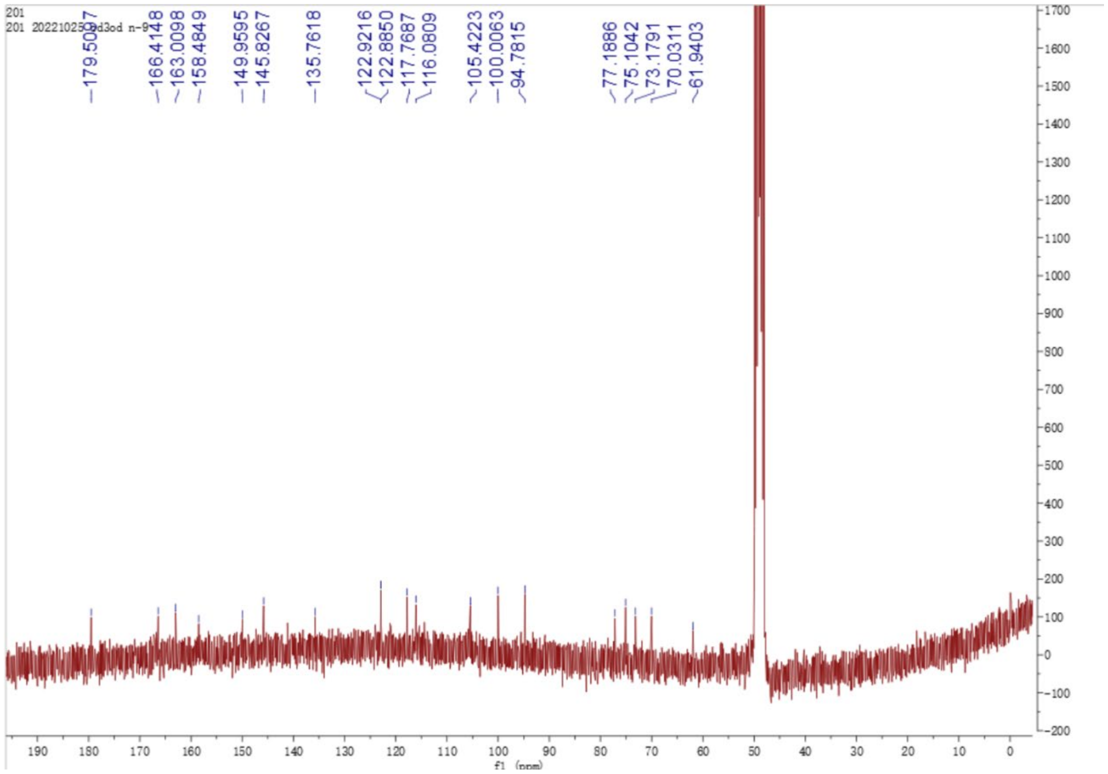

Figure S49. ^{13}C-NMR spectrum of compound **10** (75 MHz, methanol-d_4)

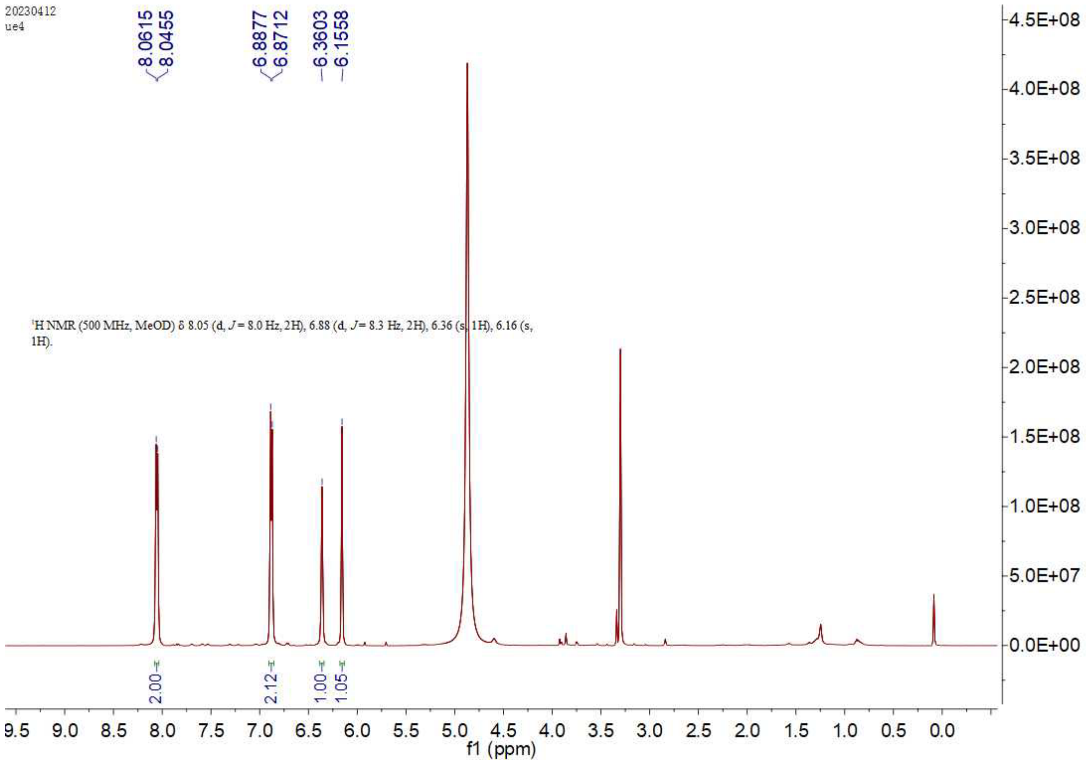

Figure S50. ^{1}H-NMR spectrum of compound **11** (300 MHz, methanol-d_4)

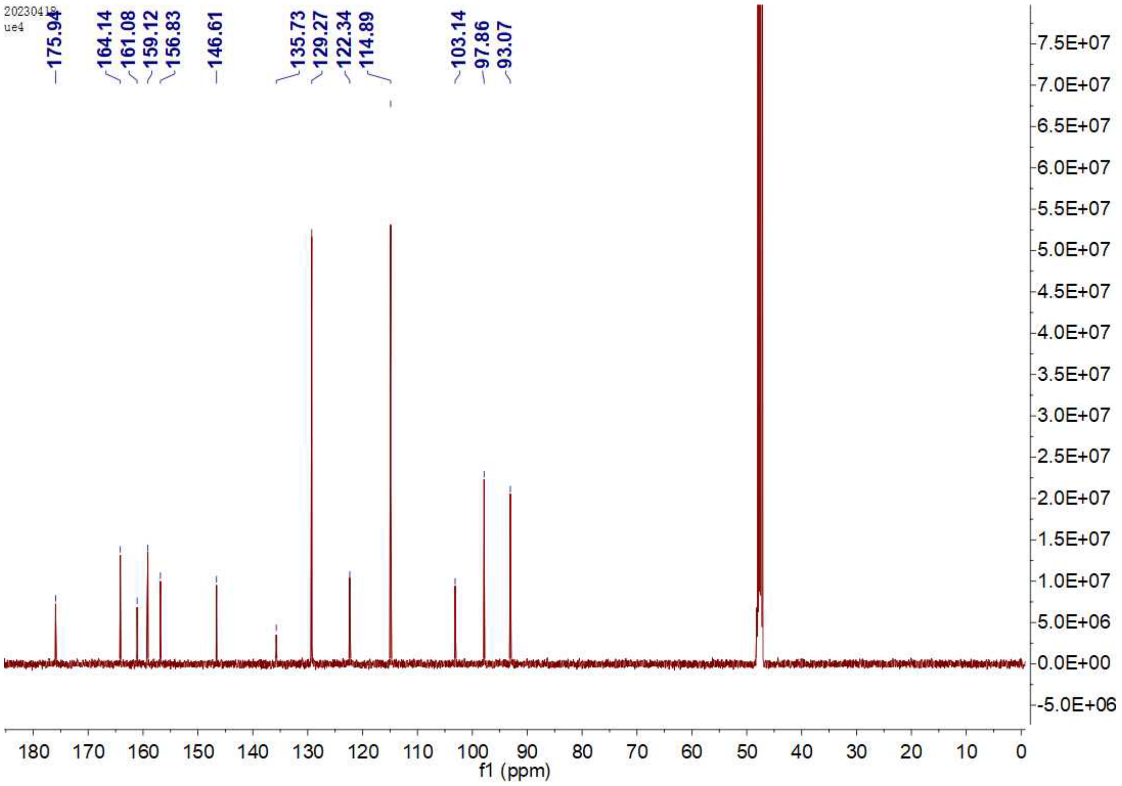

Figure S51. ^{13}C-NMR spectrum of compound **11** (75 MHz, methanol-d_4)

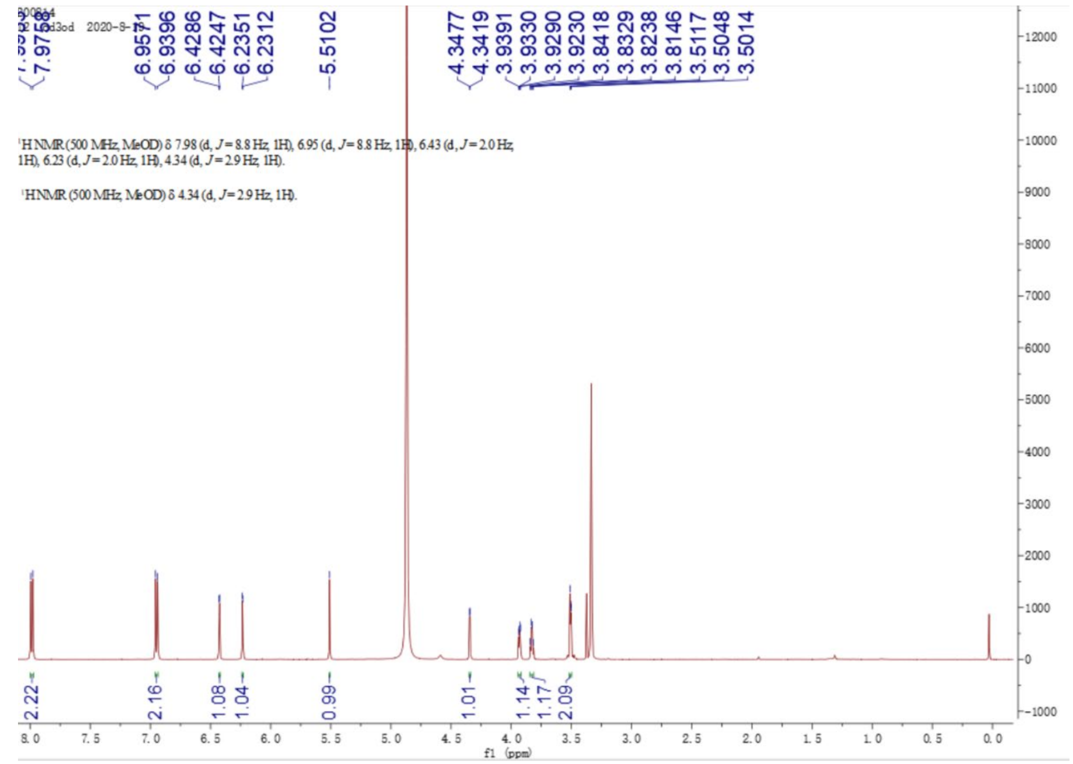

Figure S52. ^{1}H-NMR spectrum of compound **12** (300 MHz, methanol-d_4)

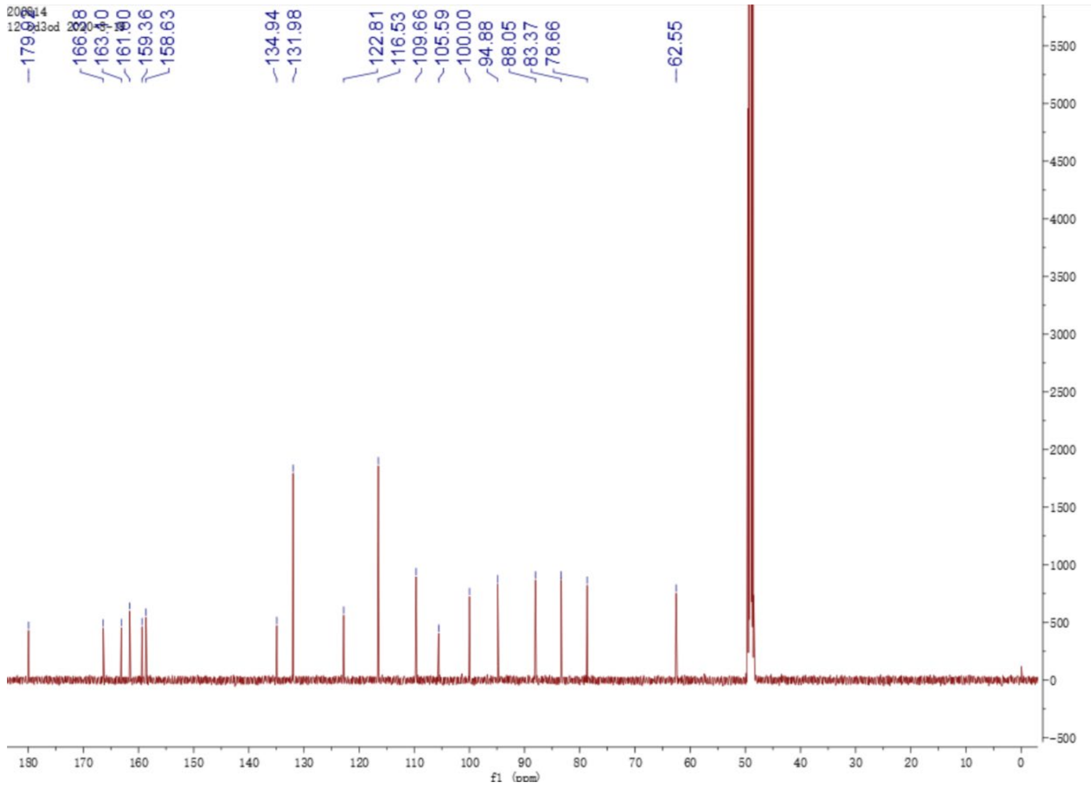

Figure S53. ^{13}C-NMR spectrum of compound **12** (75 MHz, methanol-d_4)
